# rRNA Maturation in Yeast Cells Depleted of Large Ribosomal Subunit Proteins

**DOI:** 10.1371/journal.pone.0008249

**Published:** 2009-12-11

**Authors:** Gisela Pöll, Tobias Braun, Jelena Jakovljevic, Andreas Neueder, Steffen Jakob, John L. Woolford, Herbert Tschochner, Philipp Milkereit

**Affiliations:** 1 Institut für Biochemie III, Universität Regensburg, Regensburg, Germany; 2 Department of Biological Sciences, Carnegie Mellon University, Pittsburgh, Pennsylvania, United States of America; Texas A&M University, United States of America

## Abstract

The structural constituents of the large eukaryotic ribosomal subunit are 3 ribosomal RNAs, namely the 25S, 5.8S and 5S rRNA and about 46 ribosomal proteins (r-proteins). They assemble and mature in a highly dynamic process that involves more than 150 proteins and 70 small RNAs. Ribosome biogenesis starts in the nucleolus, continues in the nucleoplasm and is completed after nucleo-cytoplasmic translocation of the subunits in the cytoplasm. In this work we created 26 yeast strains, each of which conditionally expresses one of the large ribosomal subunit (LSU) proteins. *In vivo* depletion of the analysed LSU r-proteins was lethal and led to destabilisation and degradation of the LSU and/or its precursors. Detailed steady state and metabolic pulse labelling analyses of rRNA precursors in these mutant strains showed that LSU r-proteins can be grouped according to their requirement for efficient progression of different steps of large ribosomal subunit maturation. Comparative analyses of the observed phenotypes and the nature of r-protein – rRNA interactions as predicted by current atomic LSU structure models led us to discuss working hypotheses on i) how individual r-proteins control the productive processing of the major 5′ end of 5.8S rRNA precursors by exonucleases Rat1p and Xrn1p, and ii) the nature of structural characteristics of nascent LSUs that are required for cytoplasmic accumulation of nascent subunits but are nonessential for most of the nuclear LSU pre-rRNA processing events.

## Introduction

The structural constituents of the two eukaryotic ribosomal subunits are 4 ribosomal RNAs, namely the 25S, 18S, 5.8S and 5S rRNA and more than 79 ribosomal proteins (r-proteins). They assemble in a highly dynamic process that starts with the synthesis of the precursor of 25S-, 18S- and 5.8S rRNA by RNA polymerase I and initial maturation events in the nucleolus, proceeds in the nucleoplasm and finally ends after nucleo-cytoplasmic translocation of the subunits in the cytoplasm. The 5S rRNA is synthesized by RNA polymerase III and is recruited as ribonucleoproteincomplex (RNP) together with rpL5 and rpL11 to early nuclear pre-60S particles [1].

**Figure 1:**
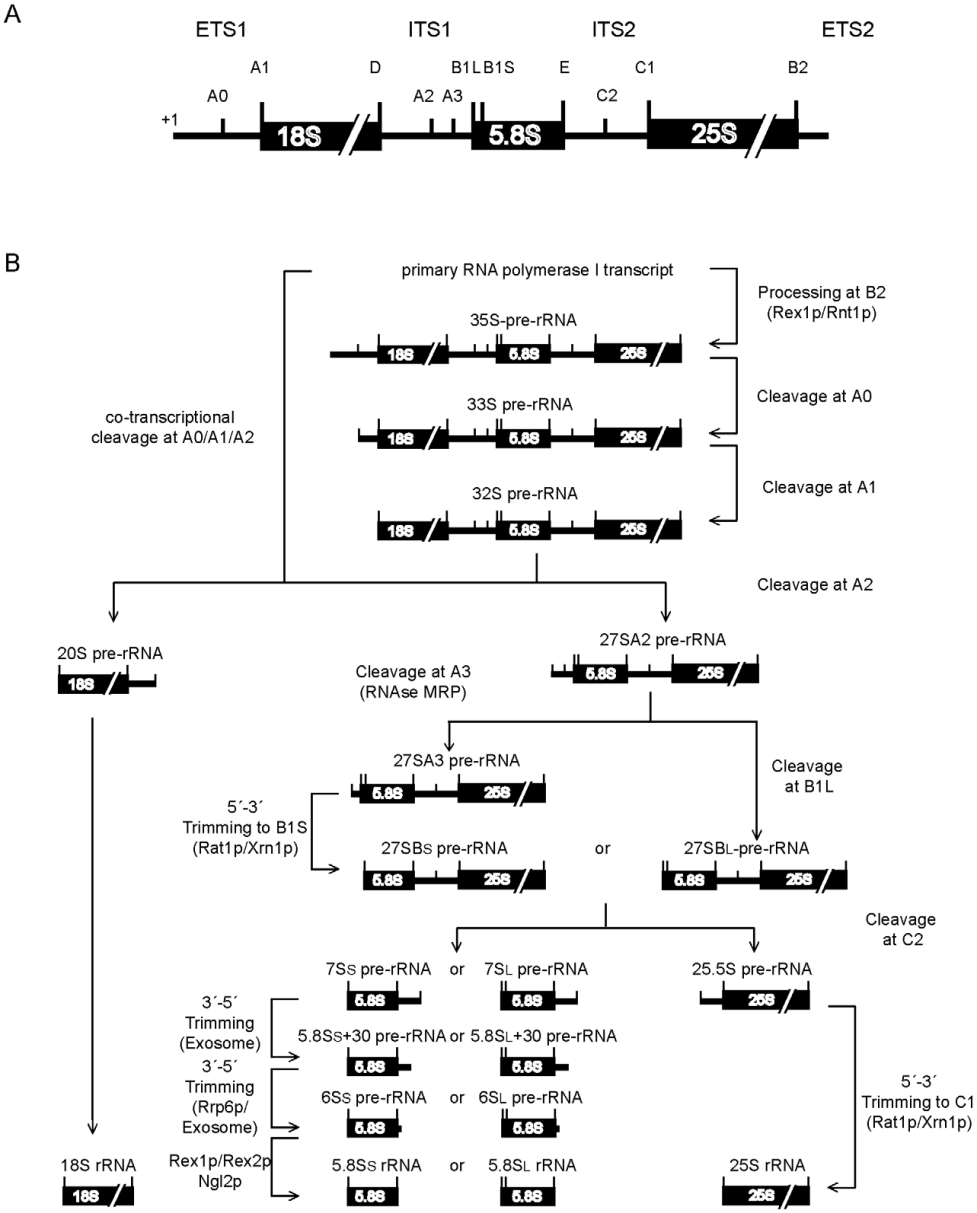
Large ribosomal subunit rRNA maturation pathways in *S. cerevisiae*. Four of the five rRNAs found in mature ribosomes are derived from transcripts made by RNA polymerase I and processed through a series of endo- and exonucleolytic reactions. In (A) External transcribed spacer regions (ETS1, ETS2), Internal transcribed spacer regions (ITS1, ITS2), the transcription start site (+1) and major rRNA processing sites of primary rDNA transcripts are indicated. In (B) major pathways of precursor rRNA processing are shown.

Genetic, biochemical and bioinformatic analysis identified more than 150 protein factors and more than 70 small nucleolar RNAs involved in eukaryotic ribosome biogenesis. The large majority of the small nucleolar RNAs, together with some of the protein factors, mediate site directed pre-rRNA modifications and a few of the protein factors have exo- and endonucleolytic activities responsible for pre-rRNA trimming and cleavage reactions (see [2] for a recent review and see Fig. 1 for an overview of pre-rRNA processing events in *S. cerevisae*). The molecular function of most of the remaining ribosome biogenesis factors is less clear. The association of many of these factors with different pre- 40S or pre- 60S ribosomal subunit assembly intermediates has been determined. Initial investigation of functions employed analyses of changes or specific blocks in pre-rRNA processing, pre-rRNA modification or nuclear export in cells in which selected factors were depleted *in vivo* or genetically inactivated. Accordingly, individual proteins or subcomplexes were suggested to participate in steps that trigger processing, modification and folding of pre-rRNA, assembly of ribosomal proteins and/or export through the nuclear pore (see [2] for a recent review). Most of these processes seem to be tightly linked [3], [4], [5]. Consequently, it is often difficult to attribute a direct molecular function to factors. In addition, 3D-localisation of ribosome biogenesis factors on the pre-ribosomes and the nature of their exact molecular interaction with (pre-) rRNA and / or r-proteins is in most cases unknown. Therefore a molecular understanding of eukaryotic ribosome biogenesis factor functions remains incomplete.

On the other hand, atomic resolution structure models of prokaryotic ribosomes and pseudo-atomic models of eukaryotic ribosomes are available (see [6] for a recent review, [7], [8]) that predict the molecular interactions of most of the eukaryotic r-proteins with rRNA in mature ribosomes. Accordingly, if particular steps in pre-rRNA processing or folding can be assigned to the presence of specific r-proteins, based on the nature of the relevant r-protein - rRNA interactions, insights may be obtained for how proteinaceous ribosomal entities influence pre-rRNA maturation. In this way, working hypotheses could be developed to implicate structural prerequisites into the pre-rRNA maturation pathway.

Previously, a comprehensive analysis of the impact of the structural organisation of r-proteins on the *in vivo* maturation and nuclear export of a ribosomal subunit was only performed for the small subunit (SSU). The three secondary structure domains of the 18S rRNA, the 5′ domain, the central domain and the 3′ domain, fold in space in two major topologically separated substructures, the head and the body domain. In *S. cerevisiae* assembly of r-proteins with the body domain of the SSU is closely related to early nuclear restricted pre-rRNA cleavage steps. R-protein – pre-rRNA assembly events in the SSU head domain have gradual impact on the cytoplasmic accumulation of nascent SSUs and the final maturation of the 3′ end of 18S rRNA [9], [5].

While six different secondary structure domains can be distinguished in 25S- and 5.8S rRNA, the large ribosomal subunit (LSU) appears in 3D structure models as “one single, gigantic domain” [10] with two lateral and one central protuberance, which contains the 5S rRNA. In remarkable contrast to SSU r-proteins, most LSU r-proteins contact more than one rRNA secondary structure domain [11] and thereby seem to contribute significantly to the formation of the LSUs compact structure through the establishment and stabilisation of a complex rRNA - r-protein and rRNA - rRNA interaction network. Accordingly, it could be assumed that - because of the “one-domain” appearance of the LSU and because of the multiplicity of interactions of LSU r-proteins - inactivation or depletion of single LSU r-proteins could result in a more common maturation phenotype rather than distinct ones. However, *in vivo* depletion of the essential yeast r-proteins rpL3, rpL5, rpL25 and rpL33 ( [12], [13], [14], [15], [16]) and the human r-proteins RPL5, RPL7, RPL11, RPL14, RPL26 and RPL35A/rpL33 [17] leads to impairment of distinguishable steps of eukaryotic pre-rRNA maturation. In addition, yeast rpL10 was suggested to play a specific role in LSU nuclear export by either recruiting the nucleo-cytoplasmic transport factor Nmd3p to nuclear LSU precursors [18] or by being involved in its release from nascent cytoplasmic LSUs [19]. Furthermore, rpL1, rpP0, rpP1, rpP2 and rpL12, constituents of the LSUs two lateral protrusions [20], [21], [22] and several nonessential LSU r-proteins, including rpL22 ( [23] and our own unpublished data), rpL41 [24], rpL24 [25], rpL31 ( [26] and our own unpublished data), rpL29 [27] and rpL39 [28] seem not to be strictly required for LSU maturation. Some of them were shown to play a role in different aspects of mRNA translation (see [29] for a review).

In this work we created 26 yeast mutant strains, each of which conditionally expresses one essential r-protein of the eukaryotic LSU. We subsequently performed a comparative analysis of phenotypes in these mutant strains and found that groups of LSU r-proteins are required for efficient progression of different steps of LSU maturation. Based on the observed phenotypes and the nature of the corresponding r-protein – rRNA interactions in current atomic LSU structure models we discuss how individual r-proteins might promote the productive processing of the major 5′ end of 5.8S rRNA precursors through protecting them from exonucleolytic degradation. In addition, the analyses presented in this work point towards structural characteristics of nascent LSUs that are required for their cytoplasmic accumulation but are nonessential for most of the nuclear LSU pre-rRNA processing events.

## Results

### Ribosomal Subunit Accumulation After Shut-Down of rpL Expression

In order to analyse *in vivo* functions of eukaryotic LSU r-proteins we created 26 yeast mutant strains conditional for expression of individual LSU r-protein genes. This set of mutants was chosen to be representative for the about 37 possibly essential r-protein genes (see introduction) and includes many genes coding for r-proteins whose bacterial homologues are located throughout the mature 50S ribosomal subunit. In each of these strains one of the LSU r-protein genes was ectopically expressed under the control of the galactose inducible *GAL1* promoter (see Materials and Methods). All 26 mutant strains could be cultivated in galactose containing medium but stopped growth when plated on medium with glucose as the sole carbon source (data not shown), indicating that each of the corresponding LSU r-proteins is essential for yeast growth. This interpretation is in agreement with previous genetic analysis done of several S. *cerevisae* r-protein gene mutants [20], [30], [13], [31], [32], [33], [34], [35], [30], [15], [36,37].

**Figure 2:**
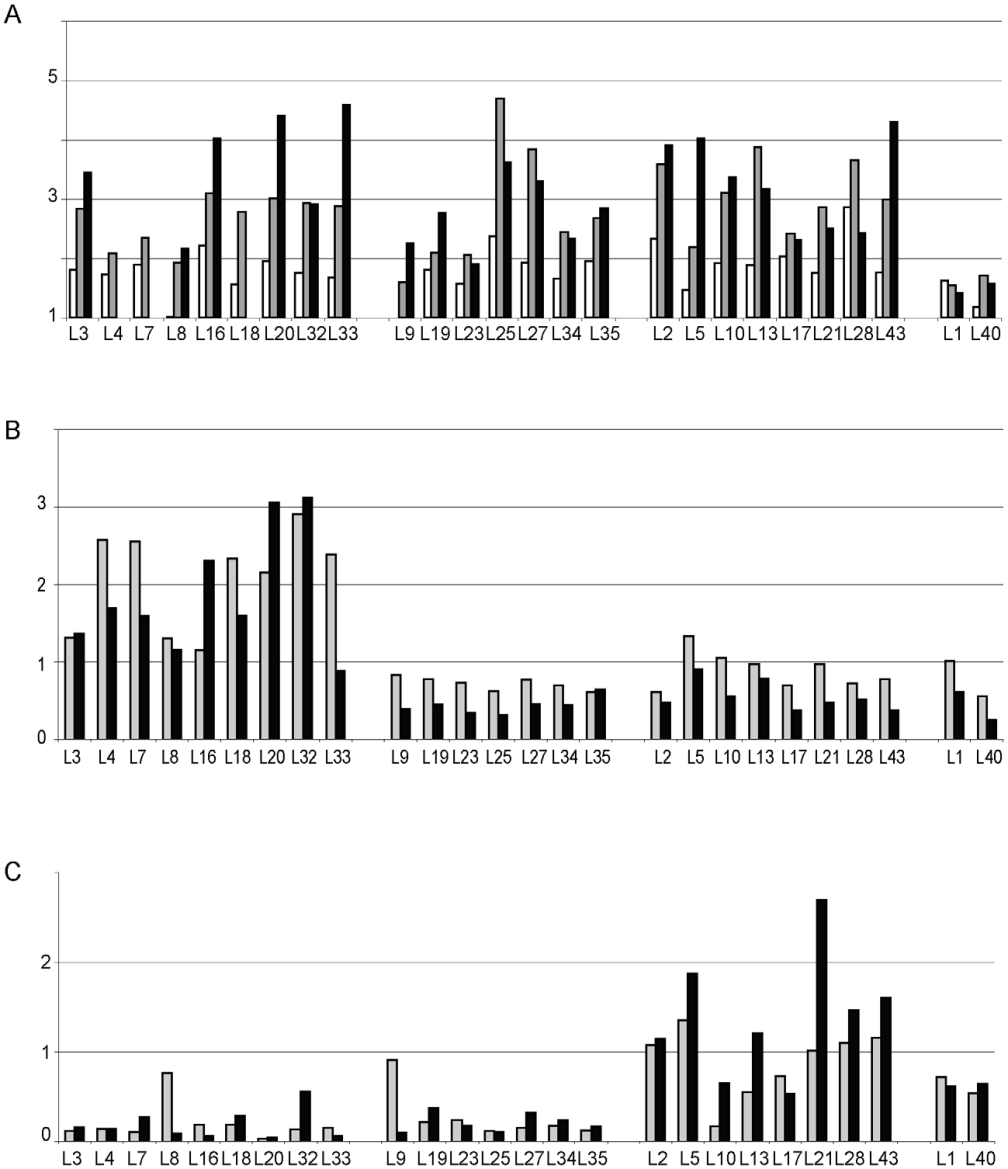
Relative accumulation of large ribosomal subunit pre-rRNAs in conditional r-protein gene mutants as indicated by Northern blotting analyses. Yeast strains expressing the indicated r-protein genes under control of the galactose-dependent *GAL1* promoter were shifted to glucose medium in A) for 2 hours (white bars), 4 hours (light grey bars) or 8 hours (dark grey bar) and in B) and C) for 2 hours (light grey bars) or 4 hours (dark grey bars). For each strain the relative accumulation of 18S rRNA over 25S rRNA (A), 27SA2 pre-rRNA over total 27S pre-rRNA (B) and 7S pre-rRNA over total 27S pre-rRNA (C) in restrictive (glucose medium) versus permissive (galactose medium) conditions was determined by Northern blotting as described in Materials and methods. These relative accumulations are indicated in the y-axis of the diagrams in A) to C). Strains are placed in groups according to rRNA processing phenotypes.

Next we analysed by Northern blotting the impact of depletion of individual essential LSU r-proteins on the accumulation of mature rRNAs. For most of the strains we observed after prolonged incubation in restrictive conditions (4 h–8 h) a significant reduction of both the LSU 25S rRNA and the SSU 18S rRNA content per OD of cells (data not shown). A similar phenotype was observed before in conditional mutants of *RPL25* and of several yeast genes coding for LSU biogenesis factors (see [14] and Discussion). In addition, all mutant strains exhibited a clear increase in the ratio of SSU 18S rRNA to LSU 25S rRNA during the first eight hours after shift to restrictive conditions (Fig. 2A). Less severe effects on ribosomal subunit balance were observed upon depletion of rpL1 and rpL40. These results suggest that *in vivo* depletion of each of the LSU r-proteins examined leads to destabilisation and degradation of the LSU and/or its precursors. rpL1 and rpL40 seem to play the least critical roles for LSU accumulation. Polysome analyses of yeast mutants of RPL1, RPL3, RPL4, RPL5, RPL8, RPL10, RPL33 or RPL40 support the latter interpretation (see Fig. S1 and [20], [38], [39], [15], [36], [12], [40], [13], [16]).

To assess more directly a role of individual LSU r-proteins in LSU production we analysed the relative changes in steady-state levels of LSU precursor rRNAs after depletion of individual LSU r-proteins by Northern blotting and primer extension experiments. Consistent with previous analyses [14], we observed in most strains under restrictive conditions a relative accumulation of 35S pre-rRNA (data not shown, see also pulse labelling experiments described below) indicating general delays in processing events in the ETS1, ITS1, and ITS2 pre-rRNA sequences (see LSU pre-rRNA processing scheme in Fig. 1).

Furthermore, mutant strains could be categorized into four major groups according to changes in levels of precursor rRNAs after r-protein depletion:(1) strains showing phenotypes in 5′ end processing of 5.8S rRNA precursors (2) strains with a delay in the endonucleolytic cleavage separating 5.8S rRNA and 25S rRNA precursors (3) strains with phenotypes in 3′ processing of 5.8S rRNA precursors, and (4) strains with no obvious major pre-rRNA processing defect.

### 5′ End Processing of 5.8S rRNA Precursors After Shut-Down of rpL Expression

**Figure 3:**
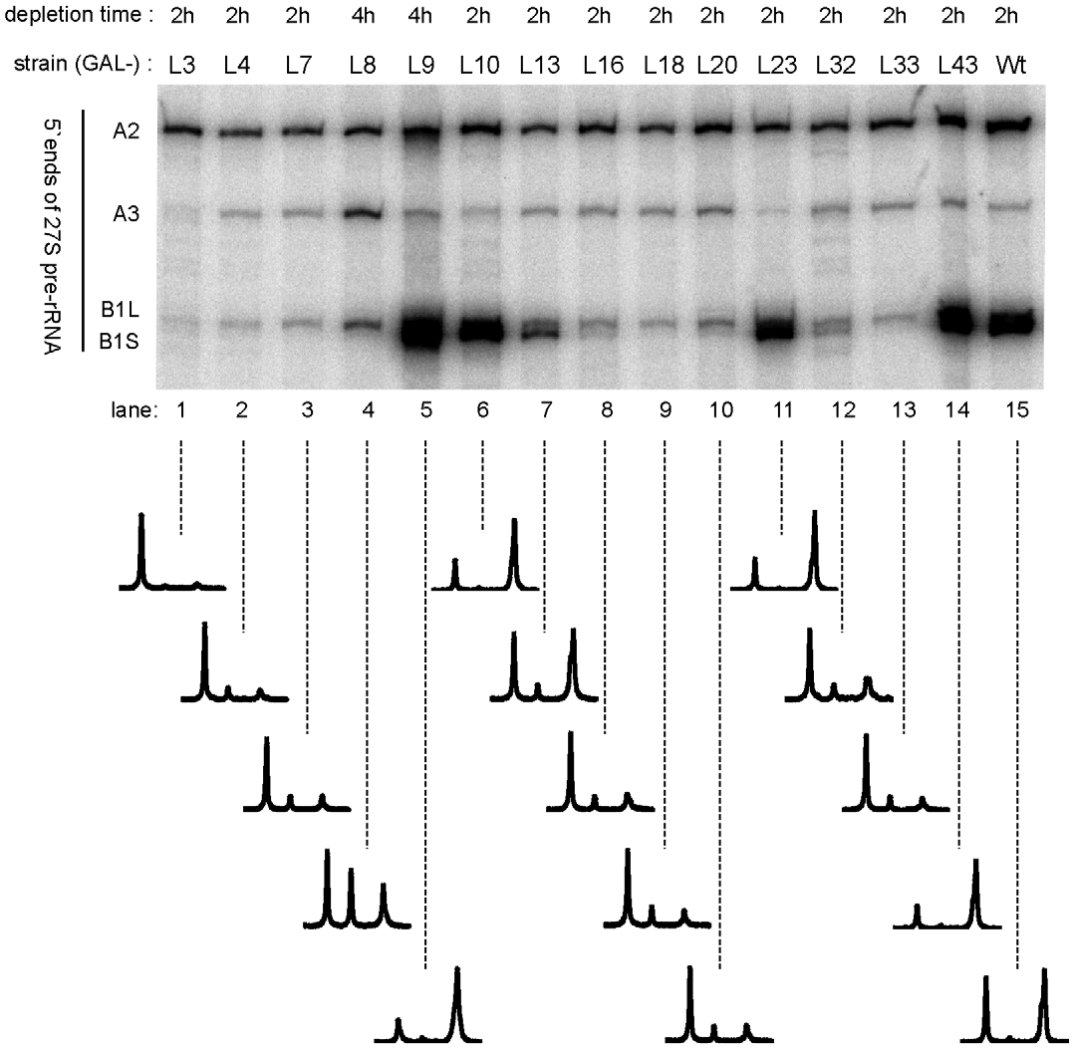
Accumulation of 27SA_2_- , 27SA_3_- , 27SB_1L_- and 27SB_1S_ pre-rRNA in conditional r-protein gene mutants as indicated by primer extension analyses. Yeast strains expressing the indicated r-protein genes under control of the galactose-dependent *GAL1* promoter were shifted for the specified times to glucose-containing medium. Primer extension analyses of LSU pre-rRNA was performed as described in Materials and methods. The diagrams in the lower panel are quantitative representations of the radioactive signals seen in the upper panel.

One group of nine mutant strains (lacking one of r-proteins rpL3, rpL4, rpL7, rpL8, rpL16, rpL18, rpL20, rpL32 or rpL33) showed an elevated ratio of 27SA2 pre-rRNA to 27SB pre-rRNA under non-permissive versus permissive conditions compared with the other strains (Fig. 2B and Fig. 3). Production of 27SA2 pre-rRNAs results in yeast from the endonucleolytic cleavage at site A2 in the ITS1 region of pre-rRNA which separates the precursor RNAs of the small and the large ribosomal subunits. Two alternative pathways exist in yeast through which 27SA2 pre-rRNAs are converted into 27SB pre-rRNAs (Fig. 1): The major pathway starts with an endonucleolytic cut at site A3 through RNAse MRP, about 70 nucleotides downstream of site A2, followed by a trimming reaction by 5′–3′ exonucleaseses Xrn1p and Rat1p [41]. These enzymes stop exonucleolytic digestion at site B1S which is the major 5′ end of mature 5.8S rRNA. The minor pathway is thought to involve an unknown endonuclease which directly cuts at site B1L, the 5′ end of about 20% of mature 5.8S rRNAs [42]. Our primer extension analyses indicated that in the nine mutant strains mentioned above pre-rRNAs with the B1S 5′ end are strongly underrepresented relative to pre-rRNAs with B1L and A2 5′ ends (Fig. 3). Accordingly, the pathway leading to B1S 5′ends of pre-rRNAs is largely non-productive when levels of any of the corresponding nine proteins are limited *in vivo*. Pre-rRNAs with B1L 5′ ends were still detected in these strains but in many cases at lower absolute levels when compared to a control strain (Fig. 3). In addition, endonucleolytic cleavage at site C2 in the ITS2 region of pre-rRNA is apparently impeded under these circumstances, since, with the exception of rpL32, only minor levels of the resulting 7S pre-rRNA (Fig. 2C) or A2-C2 fragments (data not shown) could be detected. Alltogether we conclude, that pre-ribosomes with 27SB1L pre-rRNA are still made in these strains, but are largely turned over in a non-productive way. Previously, similar pre-rRNA processing phenotypes were observed upon depletion of yeast rpL3 [12], in a yeast carrying a mutant allele of RPL33A [15], [16]and after knockdown of the mammalian homologues of rpL7 and rpL33 in human cells [17], [43].

### Endonucleolytic Cleavage between 5.8S and 25S rRNA After Shut-Down of rpL Expression

A low 27SA2 pre-rRNA to 27SB pre-rRNA ratio was observed in all other depletion strains (Fig. 2B), indicating that processing of 27SA2 pre-rRNA into 27SB pre-rRNA at site B1 could still occur. 5′ processing at site B1 in the ITS1 region of pre-rRNA is currently believed to precede the cleavage in the ITS2 region by an unknown endonclease which leads to the production of 7S pre-rRNA and 25.5S pre-rRNA, the precursors of 5.8S and 25S rRNA [44] . In seven strains (GAL-RPL9, GAL-RPL19, GAL-RPL23, GAL-RPL25, GAL-RPL27, GAL-RPL34 and GAL-RPL35) the ratio of 7S pre-rRNA to 27S pre-rRNA significantly decreased in restrictive conditions (Fig. 2C). Only minor amounts of steady state 7S pre-rRNA could be detected in this group of mutants, with the highest absolute 7S pre-rRNA level observed in the rpL19 depleted strain. Apparently, endonucleolytic cleavage in the ITS2 region of pre-rRNA at site C2, converting 27SB pre-rRNA into 7S pre-rRNA and the short lived 25.5S pre-rRNA (Fig. 1), is not completely blocked, but significantly delayed in the absence of rpL9, rpL19, rpL23, rpL25 (see also [14]), rpL27, rpL34 and rpL35.

### 3′ End Processing of 5.8S rRNA Precursors After Shut-Down of rpL Expression

**Figure 4:**
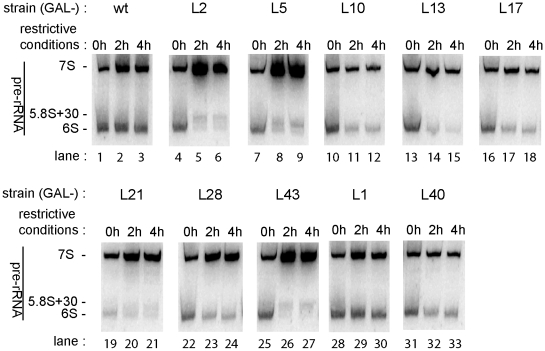
Accumulation of 3′ extended forms of 5.8S rRNA in conditional r-protein gene mutants as indicated by Northern blotting analyses. Yeast strains expressing the indicated r-protein genes under control of the galactose dependent *GAL1* promoter were shifted for the specified times to glucose containing medium. Detection of 3′ extended forms of 5.8S rRNA by northern blotting was performed as described in Materials and methods. Shown is analysis of wildtype yeast (BY4741) and of yeast strains in which separation of 5.8S rRNA precursors and 25S rRNA precursors through endonucleolytic cleavage in the ITS2 region of pre-rRNA was readily detectable.

While 27S pre-rRNA accumulated in several of the residual mutants, the relatively high 7S pre-rRNA to 27S pre-rRNA ratio observed after shifting these ten strains (GAL-RPL1, GAL-RPL2, GAL-RPL5, GAL-RPL10, GAL-RPL13, GAL-RPL17, GAL-RPL21, GAL-RPL28, GAL-RPL40, and GAL-RPL43) to restrictive conditions (Fig. 2C) argues that cleavage at site C2 still takes place in precursor LSUs lacking the corresponding ribosomal proteins. It had been shown previously that 3′ extended forms of 5.8S rRNA (Fig. 1) strongly accumulate in yeast strains carrying deletions of the nonessential genes encoding the 3′–5′ exonucleases Rrp6p, Rex1p, Rex2p, Rex3 or the endonuclease Ngl2p [45], [46], [47]. Some of these 3′ extended forms of 5.8S rRNA seem to be able to carry out the essential functions of 5.8S rRNA in cytoplasmic ribosomes ([45], [46] and discussion therein, [48]). In none of the mutant strains we could detect a comparably strong increase of these intermediates or of the short lived 25.5S pre-rRNA (Fig. 4 and data not shown) which is stabilised after inactivation of the two 5′–3′ exonucleases Rat1p and Xrn1p [49]. On the other hand, upon depletion of rpL2, rpL5 and rpL43 and, to a minor degree rpL21, we observed a pronounced relative accumulation of 7S pre-rRNA versus 5.8S+30 pre-rRNA and of 5.8S+30 pre-rRNA versus 6S pre-rRNA (Fig. 4). This indicates a specific delay of exosome-mediated 3′ processing of 5.8S rRNA. A previously performed siRNA mediated knock-down of L5 resulted in similar pre-rRNA processing phenotypes in cultivated human cells [17]. Upon depletion of rpL1, rpL40, rpL10, rpL13, rpL17 and rpL28, we did not detect major pre-rRNA processing phenotypes except for the generally observed accumulation of 35S pre-rRNA (see above). Apparently, most steps of LSU rRNA maturation can occur in pre-ribosomes lacking these r-proteins.

### Dynamics of Maturation and Cytoplasmic Accumulation of Nascent rRNAs and tRNAs After Shut-Down of rpL Expression

**Figure 5:**
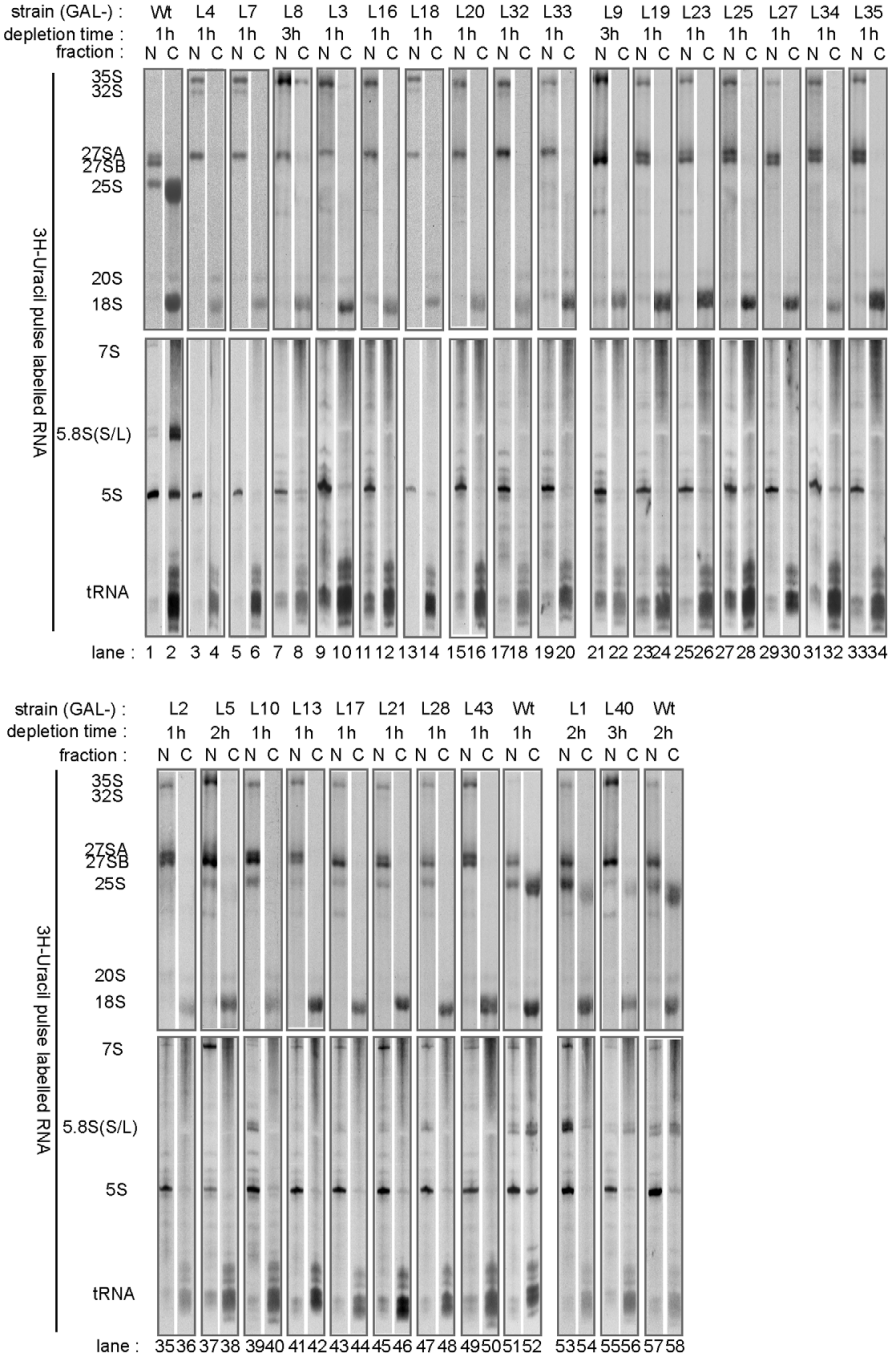
Neo-synthesis and intracellular transport of tRNA and rRNA in conditional r-protein gene mutants as indicated by metabolic RNA labelling and nucleo-cytoplasmic fractionation. Yeast strains expressing the indicated r-protein genes under control of the galactose-dependent *GAL1* promoter were shifted for the specified times to glucose-containing medium. Pulse-labelling of total RNA (15 minutes) followed by nucleo-cytoplasmic fractionation and visualisation of newly synthesised RNA contained in nuclear (N) and cytoplasmic (C) fractions was performed as described in Materials and methods. Two point five times more RNA of nuclear than of cytoplasmic fractions was analysed.

To analyse more directly the efficiency and dynamics of LSU pre-rRNA processing and of the transport of precursor LSUs from the nucleus to the cytoplasm in the mutant strains, we performed RNA pulse labelling experiments, followed by nucleo-cytoplasmic fractionation. As depicted in Fig. 5, lanes 1 and 2, pulse labelled newly synthesized LSU pre-rRNAs (35S-, 27SA-, 27SB- and 7S pre-rRNA) could be detected in wildtype cells exclusively in the nuclear fraction while newly synthesized 25S rRNA and 5.8S rRNA accumulated in the cytoplasmic fraction. Newly synthesized 5S rRNA was detected in apparent excess over 5.8S rRNA in nuclear fractions while cytoplasmic fractions contained similar amounts of both pulse labelled LSU rRNA species. These results agree with conclusions drawn from similar experiments that in yeast cells nascent LSUs containing 25S rRNA, 5.8S rRNA (or its functional equivalent 3′ extended forms, see above) and 5S rRNA are adequate substrates of the nuclear export machinery [50], [51], [52].

Newly synthesized 25S rRNA and 5.8S rRNA or their precursors did not accumulate in the cytoplasm of any of the analysed mutants, except pGAL-RPL1 and pGAL-RPL40. On the other hand, in all strains synthesis and nuclear export of tRNA and (pre-)18S rRNA clearly took place during the period of the ^3^H-uracil pulse (Fig. 5) even if nuclear accumulation of newly synthesized 35S rRNA, a putative common precursor of 18S rRNA, 25S rRNA and 5.8S rRNA (see Fig. 1 and above), was evident. These observations indicate that limited expression of individual LSU r-proteins primarily results in a rather specific and predominant effect on LSU production. Interestingly, in many mutant strains we observed substantial production of nuclear 5S rRNA and detected some minor amount of newly synthesized 5S rRNA in the cytoplasm (Fig. 5, lanes 8,10,12,14,26,28,30,32), leaving open the possibility that in yeast 5S rRNA containing particles can translocate to the cytoplasm independently of most of the residual LSU components (see also discussion in [51]).

In strains in which rpL3, rpL4, rpL7, rpL8, rpL16, rpL18, rpL20, rpL32 and rpL33 were depleted, the 35S- and 27SA pre-rRNAs were the only prevalent newly synthesized precursors of the 25S rRNA and 5.8S rRNA (Fig. 5 lanes 3,5,7,9,11,13,15,17,19). In strains lacking rpL9, rpL19, rpL23, rpL25, rpL27, rpL34 or rpL35, 27SB pre-rRNAs were clearly produced during the time of pulse labelling, but newly synthesized 7S pre-rRNA and 25S rRNA/25.5S rRNA were hardly observable (Fig. 5 lanes 21,23,25,27,29,31,33). Altogether these data indicate that assembly of rpL3, rpL4, rpL7, rpL8, rpL16, rpL18, rpL20, rpL32 and rpL33 is required for productive generation of 27SB pre-rRNA from 27SA pre-rRNA while assembly of rpL9, rpL19, rpL23, rpL25, rpL27, rpL34 and rpL35 are important for efficient conversion of 27SB pre-rRNA into 7S pre-RNA and 25.5S pre-rRNA containing pre-LSU's through endonucleolytic cleavage in the ITS2 pre-rRNA region at site C2. In addition, the absence of all these ribosomal proteins seems ultimately to lead to nuclear degradation of nascent LSUs.

In strains GAL-RPL1, GAL-RPL2, GAL-RPL5, GAL-RPL10, GAL-RPL13, GAL-RPL17, GAL-RPL21, GAL-RPL28, GAL-RPL40 and GAL-RPL43, for which the steady state analyses suggested that cleavage in the ITS2 region of pre-rRNA still occurs to some extent, neo-synthesis of the resulting nuclear 7S pre-rRNA (Fig. 5, lower panel, lanes 35, 37, 39, 41, 43, 45, 47, 49, 53, 55) and, at least for strains GAL-RPL5, GAL-RPL10, GAL-RPL13, GAL-RPL17, GAL-RPL21, GAL-RPL28, GAL-RPL1 and GAL-RPL40, neo-synthesis of nuclear 25.5S/25S rRNA (Fig. 5, upper panel, lanes 37, 39, 41, 43, 45, 47) was detectable. In addition, in strains GAL-RPL10, GAL-RPL13, GAL-RPL17, GAL-RPL21 and GAL-RPL28, but not in strains GAL-RPL2, GAL-RPL5 and GAL-RPL43, some production of short lived nuclear pre-LSUs containing 6S/5.8S rRNA was evident, which apparently were precluded from translocation through the nuclear pores (Fig. 5, compare lanes 39, 41, 43, 45 and 47 with lanes 35, 37 and 49). In agreement with previous analyses [13], [1] demonstrating a role for yeast rpL5 in 5S rRNA stability and in recruitment of an 5S rRNA – rpL5 – rpL11 RNP into the LSU precursors, in strain pGAL-RPL5 accumulation of newly synthesized 5S rRNA was reduced compared to tRNA accumulation (Fig. 5 lower panel, compare lanes 37 and 38 with lanes 57 and 58). In conclusion, these analyses suggest that in the absence of rpL2 and rpL43 as in the absence of rpL5, and consequently the 5S rRNA – rpL5 – rpL11 RNP, unstable nuclear restricted pre-LSUs containing 25S rRNA and 7S pre-rRNA are made which are inefficient substrates for consequent exosome mediated pre-rRNA processing events. On the other hand, when expression of genes coding for rpL10, rpL13, rpL17, rpL21 and rpL28 is shut down, the cellular production of nuclear pre-LSUs containing matured rRNAs is not completely blocked. However, the data suggest that these pre-LSUs lacking rpL10, rpL13, rpL17, rpL21 or rpL28 are largely restricted to the nucleus and are finally degraded.

In strains depleted of rpL40 or rpL1 significant amounts of newly synthesized cytoplasmic LSUs containing 25S rRNA, 5S rRNA and 5.8S rRNA were detected. Nevertheless, cytoplasmic LSU accumulation was slightly reduced compared to a wildtype strain (Fig. 5, compare lanes 54 and 56 with lane 58). In agreement with this, both strains showed comparably low, but detectable ribosomal subunit imbalance phenotypes in steady state analyses after shift to restrictive conditions (see above, Fig. 2A, Fig. S1). Interestingly, in *rpl1* but not in *rpl40* mutant the ratio between newly synthesized nuclear and cytoplasmic 25S and 5.8S rRNA was clearly increased when compared to wildtype cells (Fig. 5, compare lanes 53 and 54 with lanes 55–58), indicating a delay of nucleo-cytoplasmic transport of pre-LSUs which fail to assemble rpL1.

## Discussion

In the work presented here we created 26 yeast strains each of which conditionally expresses one of the 46 ribosomal proteins of the large ribosomal subunit. None of the mutant strains exhibited significant growth under restrictive conditions, indicating that production of the majority of LSU r-proteins is essential for yeast growth. After long term *in vivo* depletion of most LSU proteins, we observed not only a decrease in cellular content of LSUs and a resulting ribosomal subunit imbalance but also a clear, albeit less pronounced decrease in the amount of SSUs per cell. The latter phenotype was seen before in conditional mutants of RPL25 and of several LSU biogenesis factors (see [14] and discussion therein). On the other hand, soon after shifting to restrictive conditions, we could see specific effects on production of new LSUs in most conditional LSU r-protein gene mutant strains whereas biogenesis of SSUs remained largely unaffected. Therefore we conclude that a primary effect of shortage of LSU r-protein expression is on pre-LSU maturation.

In most cases, shutdown of individual LSU r-protein gene expression led to rather strong defects in different, specific steps of LSU maturation, namely 5′ maturation of 5.8S pre-rRNA, endo-and exonucleolytic processing of the ITS2 region of pre-LSU rRNA and cytoplasmic accumulation of LSU precursors. Despite the fact that current 3-D models of the LSU clearly indicate a complex network of interactions between its structural components, individual groups of LSU r-proteins seem to have specific impact on different aspects of rRNA maturation.

In principle, various molecular functions in rRNA maturation and transport can be envisioned for r-proteins as for ribosome biogenesis factors. They could have intrinsic exo- or endonucleolytic activity required for rRNA maturation or facilitate by themselves passage through nuclear pores. They could directly mediate the interaction of pre-LSUs with rRNA maturation/transport factors, as was suggested for rpL10 [18] or they could be involved in building up local or global structures that allow the interaction of rRNA maturation/transport factors. In addition they could trigger the release of maturation/transport factors from pre-LSUs, as might be the case for the nonessential rpL24 [53] and for rpL10 [19], or they could be involved in protecting pre-LSUs from degradation by endo- and exonucleases.

Structural models may help to predict molecular functions of r-proteins: Primary rRNA maturation/transport phenotypes observed in strains carrying conditional r-protein-gene mutants can be compared with the exact positioning of the corresponding proteins in current atomic resolution structure models of eukaryotic ribosomes.

**Figure 6:**
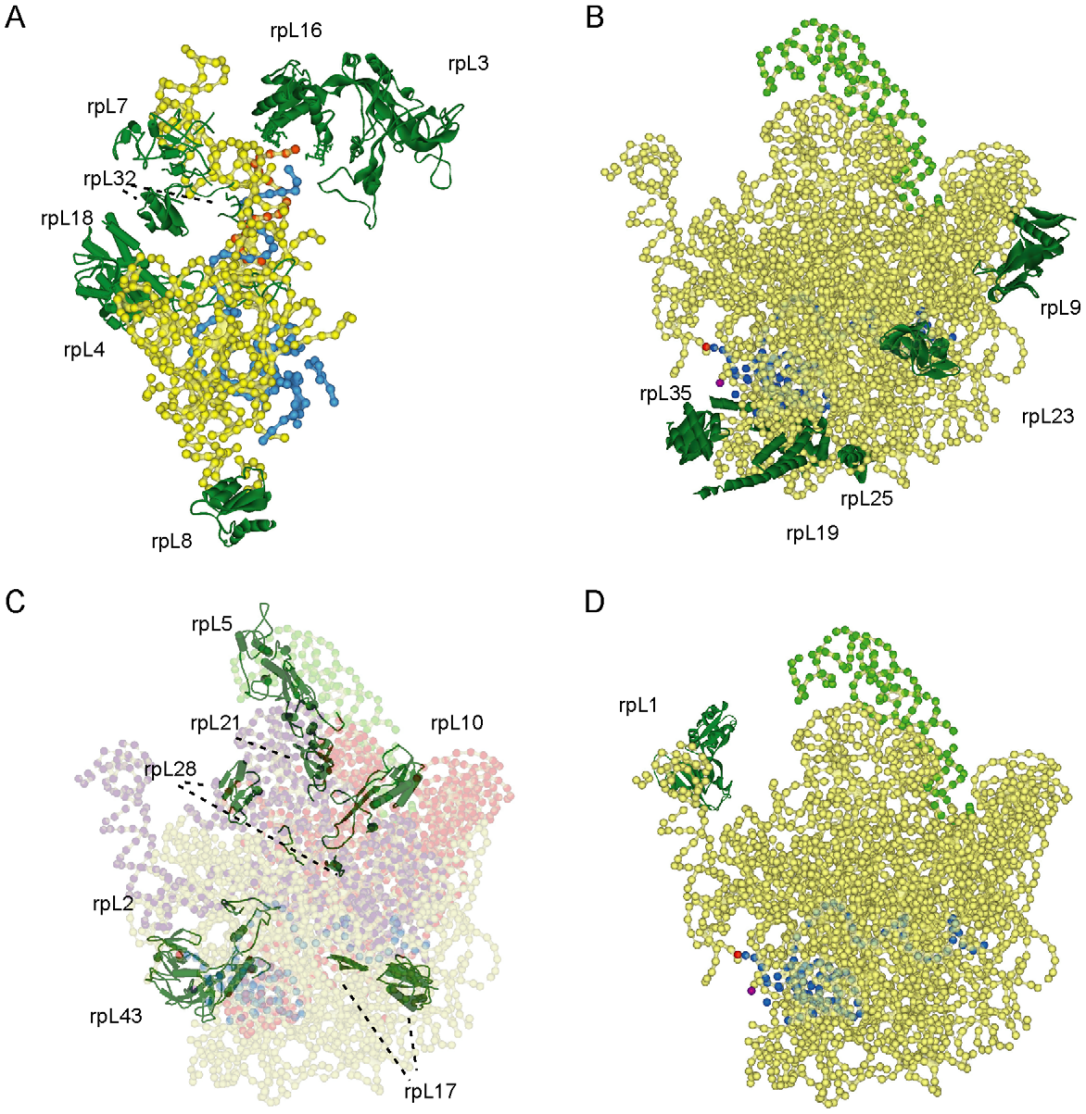
rRNA – r-protein interactions as indicated by atomic resolution structure models of the eukaryotic mature cytoplasmic LSU. PDB file 2ZKR from [7], based on a 8.7 A electron cryomicroscopy map of the mammalian ribosome in which rRNA and homology models of r-proteins were docked, was used in (A) – (D). The phosphate backbone of (parts of) LSU rRNA (in yellow, if not stated otherwise) and ribbon representations of 20 r-proteins with currently known 3D-localisation are shown (dark green). In (A) domain I and the 5′ region of domain II of LSU rRNA are visualised with the 5.8S rRNA in blue and a part of domain II forming a helical structure with the 5′ end of 5.8S rRNA in red. In (B) – (D) the LSU is seen in the crown view with the L1 stalk on the left, the (L7/L12-)Phospho-stalk on the right and the 5S rRNA in light green. In (B) - (D) 5.8S rRNA is highlighted in blue, its 3′ end in red and the 5′ end of 25S rRNA in violet. In (C) rRNA is shown transparent, with LSU rRNA domain V in violet and LSU rRNA domain II in red.

Down-regulation of expression of r-proteins rpL3, rpL4, rpL7, rpL8, rpL16, rpL18, rpL20, rpL32 and rpL33 resulted in inefficient production of pre-rRNAs with a matured 5′ end of 5.8S pre-rRNA. More specifically, the maturation pathway leading in yeast to the major 5′ end of 5.8S pre-rRNA was strongly affected in these mutant strains. This pathway is initiated by an endonucleolytic cut about 80 nucleotides upstream of the 5′ end of 5.8S rRNA at site A3 and involves then an exonucleolytic trimming reaction mediated by the general 5′–3′ exonucleases Rat1p/Xrn1p. These enzymes stop exonucleolytic digestion at site B1S which is the 5′ end of about 80% of mature 5.8S rRNA in wildtype conditions. Detailed analyses of current 3D-folding models of eukaryotic LSU rRNAs [7] suggests that the 5′end of 5.8S rRNA forms an extended secondary structure network involving a part of domain II of 25S rRNA and that formation of these interactions requires correct folding of domain I and domain II sequences positioned inbetween these two elements (Fig. 6A). Interestingly, rpL3, rpL4, rpL7, rpL8, rpL16, rpL18, rpL20, rpL32 and rpL33 are LSU r-proteins that contact this area of LSU rRNA domains I and II or are closely positioned near the 5′ end of 5.8S rRNA (Fig. 6A). Thereby it seems plausible that 1) these r-proteins help establish the 3-dimensional organisation of LSU rRNA domains I and II leading to the extensive rRNA-rRNA interaction network at the 5′ end of 5.8S rRNA, and 2) the establishment of rRNA-rRNA interactions at the 5′ end of 5.8S rRNA is important to restrict the exonucleolytic action of Rat1p/Xrn1p to correctly trim LSU pre-rRNAs rather than allowing Rat1p/Xrn1p to degrade LSU pre-rRNAs.

Down-regulation of expression of another group of LSU r-proteins resulted in a pronounced delay of endonucleolytic cleavage in the ITS2 region of pre-rRNA at site C2. According to current 3D-models of the eukaryotic LSU rpL19, rpL25 and rpL35 are positioned at the bottom of the LSU, and rpL23 and rpL9 on an axis spanning from there towards the base of the (L7/L12-)phospho-protein stalk (see Fig. 6B). According to secondary structure models [54], [55], [56] the ITS2 region of pre-rRNA is predicted to fold in several helical segments with site C2 about 135 nucleotides away from the 5.8S rRNA 3′ end and 100 nucleotides away from the 25S rRNA 5′ end. How the ITS2 region of pre-rRNA folds in space and how it orients toward other parts of the LSU precursor particle is currently not known.

We also found in this study that the expression of another group of LSU r-proteins was specifically required for efficient final 3′ maturation of 5.8S rRNA precursors (rpL5, rpL21, rpL2, rpL43) and/or productive nuclear export of LSU precursor particles (rpL10, rpL13, rpL17, rpL21, rpL28). In current 3D models these proteins are distributed all over the LSU (Fig. 6) with one cluster around the LSUs central protrusion (rpL5, rpL21, rpL10), with rpL28 near the L1 stalk, rpL17 near the exit tunnel and rpL2 and rpL43 at the subunit interface close to the 3′ end of 5.8S rRNA. Several of these proteins are prototypic examples of r-proteins that fold in a globular domain with a protruding extension (rpL2, rpL28, rpL17, rpL5). These extensions are characterised by a high content of basic aminoacids that reach inside the RNA core of the LSU and are responsible for a disproportionally high amount of RNA-protein interactions found in the LSU [11]. Our data suggest, that rpL2, rpL28, rpL17 and rpL5, which are as mentioned above examples of r-proteins carrying these extension domains, are not strictly required for all (rpL28, rpL17, see Fig. 5 lanes 47–48 and lanes 43–44) or most (rpL2, rpL5, see Fig. 5 lanes 35–38) of the LSU pre-rRNA processing steps. In support of this, recent work showed that in prokaryotes the extension domains of some r-proteins, including the rpL17 homologue L22, are not required for *in vivo* assembly of ribosomal subunits [57], [58].

A remarkable characteristic of the r-proteins identified here to be involved in final nuclear steps of pre-rRNA maturation and/or cytoplasmic accumulation of LSUs is that most of them (rpL2, rpL5, rpL10, rpL17, rpL21 and rpL28) interact both with domains II and V of LSU rRNA [10]. rpL43, the only exception, interacts with LSU rRNA domain II, not with domain V but, on the other hand, is in close contact with rpL2. LSU rRNA domains II and V, together with rpL10, rpL21 and rpL7 build an interaction platform for the 5S rRNA - rpL5 - rpL11 RNP, the major constituent of the LSUs central protruberance. Therefore it seems, that correct positioning of the 5S rRNA – rpL5 – rpL11 RNP in the LSU is specifically required for efficient final nuclear 3′ processing of 5.8S pre-rRNA and / or cytoplasmic accumulation of nascent LSUs. Future analyses will have to show whether in this group of conditional r-protein-gene mutants the 5S RNP is physically excluded from newly synthesised LSUs as was observed before in cells depleted *in vivo* for rpL5, rpL11 or one of the ribosome biogenesis factors Rpf2p and Rrs1p [1]. In any case, inspection of LSU 3D structure suggests that the 5S RNP and the extended protein folds found in rpL2, rpL17 and rpL28 promote the correct positioning of LSU rRNA domains towards each other. Apparently, late nuclear pre-rRNA maturation events and productive nucleo-cytoplasmic translocation correlate with the establishment of a highly ordered structural organisation of the nascent LSUs that is not strictly required for other nuclear steps of LSU pre-rRNA maturation.

The observation that nuclear export of nascent LSUs was not blocked, but detectably delayed when expression of rpL1 was downregulated, furthermore suggests that also local changes in nascent LSU structure can detectably affect its nucleo-cytoplasmic translocation efficiency: rpL1 is the only protein constituent of one of the LSUs two lateral protruberances (Fig. 6). Lowered expression of rpL1 does neither lead to significant pre-rRNA processing phenotypes (see above, [20]) nor does the omission of its prokaryotic homologue L1 affect the assembly of the residual LSU in *in vitro* reconstitution experiments with purified prokaryotic LSU components [59].

Altogether the LSU pre-rRNA maturation phenotypes observed here in strains conditionally expressing LSU r-proteins do not match exactly most of the ones observed in yeast strains in which components of the endo- and exonucleases involved in LSU pre-rRNA processing, namely RNAse MRP [60], [61], Rat1p, Xrn1p [49], [41], Rnt1 [62], Ngl2p [47], Rex1p, Rex2p, Rex3p [46] or the exosome [45], [63], were inactivated or *in vivo* depleted. Strikingly, individual and/or combinational deletions of several of the genes coding for RNAses involved in pre-rRNA processing are not lethal (Xrn1p, Rnt1p, Ngl2p, Rex1p, Rex2p, Rrp6) but lead to rather strong accumulation of LSUs containing immature rRNA precursors [46], [62], [60], [47], [45], [63]. In contrast, *in vivo* depletion of most of the yeast LSU r-proteins was lethal and resulted in nuclear restricted newly synthesized LSUs which contained partial or fully processed rRNA and were ultimately substrates for degradation. Similar phenotypes were observed in a large number of conditional alleles of LSU biogenesis factors whose primary structure does not contain obvious indications for their direct role as pre-rRNA processing enzymes ( see [2] for a review). Whether and how some of these factors promote the assembly of r-proteins [64], [1], [65], [66], [67]remains in most cases to be answered. We assume that this work can help in future studies to understand better the interplay of ribosome assembly factors, individual transient or non-transient assembly events on nascent subunits and LSU rRNA precursor maturation in eukaryotic cells.

## Materials and Methods

### Yeast Cell Culture, Strain Construction and Plasmid Construction

Standard protocols were followed for cultivation, transformation, mating, sporulation, preparation of genomic DNA, and tetrad dissection of yeast [68]. Selection of 5-FOA resistant clones and plasmide shuffling experiments were carried out on YNB supplemented with glucose or galactose, respectively and the amino acids required, in the presence of 1g/l of 5-FOA (Toronto Research). The general strategy for construction of conditional rpL-gene mutants was as described in [5] or [69]. For a complete list of the resulting strains, their genotypes and the exact description of individual strain and plasmid construction see Tables in Figures S2, S3, S4.

### Steady-State Analysis of (pre-)rRNA

For steady-state analyses of different (pre-) rRNA species, yeast strains were grown in galactose containing medium (YPG) at 30°C, then centrifuged and resuspended in galactose (YPG) or glucose-containing medium (YPD) to an OD of 0.3. Cells were incubated at 30°C and at the indicated timepoints (2 h for YPG cultures) 1.5 OD of cells were harvested and washed in ice cold water. RNA was extracted by hot acidic phenol–chloroform treatment [70]. Primer extension analyses were done as described in [71] using a primer complementary to the C1-C2 region of pre-rRNA (O211: 5′-GAACATTGTTCGCCTAGA-3′). Northern blotting analyses after RNA separation on formaldehyde/MOPS agarose gels (Figure 2) or Urea/TBE/Polyacrylamid gels (Figure 4) were done essentially as described in [72]. Hybridization with probes was performed in 50% formamide/5x SSC/0,5% SDS/5x Denhardt's solution at 30°C (25°C for probe O1935) with the following ^32^P-labelled probes: O205 (18S-rRNA): 5′-CATGGCTTAATCTTTGAGAC-3′; O212 (25S-rRNA): 5′-CTCCGCTTATTGATATGC-3′; O210 (E-C2 region of pre-rRNA for detection of 7S pre-rRNA and total 27S pre-rRNA): 5′-GGCCAGCAATTTCAAGTTA-3′; O1935 (5′of pre-rRNA ITS2 region for detection of 7S pre-rRNA, 5.8S + 30 pre-rRNA and 6S pre-rRNA): 5′-TGAGAAGGAAATGACGCT-3′ [63], O207 (A2-A3 region of pre-rRNA for detection of 27SA2 pre-rRNA): 5′-TGTTACCTCTGGGCCC-3′. The blots were washed twice for 15 min with 2x SSC at 30°C (25°C for probe O1935). Labeled (pre-)rRNA signals were detected using a Phosphor Imager FLA3000 (Fujifilm) and data were quantified using MultiGauge V3.0 (Fujifilm).

### Metabolic Labelling of RNA and Nucleo-Cytoplasmic Fractionation

Metabolic labeling of total yeast RNA with ^3^H-uracil and subsequent nucleo-cytoplasmic fractionation was done essentially as described in [5], [50]. In brief, 30 optical density units of yeast cells logarithmically growing in YPG were harvested and washed twice in water and were resuspended in 50 ml of buffer Z (2 mM MgCl_2_, 10 mM sodium citrate pH 7.5, 120 g/l mannitol, 9 mM beta-mercapto-ethanol). Cells were incubated for 30 min at 37°C before 2 mg of zymolyase 100T (Seikagagu) suspended in buffer Z was added. After 15 min incubation at 37°C the suspension was cooled down on ice, subsequently washed twice in buffer Z at 4°C , suspended in 25 ml YPD with 120 g/l mannitol and incubated for 35 minutes at 30°C under mild movement. Cells were centrifuged for 8 min at 3000 g, washed in 0.5 ml buffer R (20 g/l glucose, 10 g/l bactopeptone, 6 g/l malt extract, 0.1 g/l yeast extract, 3.8 g/l magnesium acetate (*4H_2_O), 120 g/l mannitol) and suspended in 0.2 ml buffer R. 20 ul [5,6-^3^H] uracil (1 mCi/ml, GE-Healthcare) was added and after 15 min incubation at 30°C cells were cooled down on ice and centrifuged for 3 min. at 3000 rpm at 4°C in a table top centrifuge. The supernatant was discarded and the cells were suspended in 0.7 ml cold buffer P (8% polyvinylpyrrolidon av. Mr = 40000, 1 mM MgCl_2_, 20 mM potassium phosphate pH 6.5, 10 mM EDTA) containing 0.03%(w/v) Triton-X100. Cells were broken on ice by about 20 strokes in a Douncer (25–75 micrometer clearance, 1 ml, Wheaton) and 0.7 ml of buffer P containing 0.6M sucrose was added. The suspension was layered on 0.5 ml of buffer P containing 0.45M sucrose and centrifuged for 10 min at 3900 g in a swing out rotor at 4°C. RNA was extracted by hot acidic phenol/chloroform treatment [70] from 0.2 ml of the upper layer ( =  fraction enriched for cytoplasmic material) and 0.1 ml of the material that was spun down to the bottom of the tubes (enriched for nuclear material) and suspended in 0.3 ml of buffer P containing 0.45M sucrose. Twenty percent of the extracted RNA was separated on formaldehyde/MOPS agarose gels (Figure 2) and urea/TBE/polyacrylamid gels essentially as described in [72], transferred to membranes and ^3^H labelled RNA was visualised by fluorography (large RNAs with En3Hance, Perkin Elmer and small RNAs with TranScreen-LE, Sigma-Aldrich). Processing and analysis of newly synthesised RNA contained in nuclear or cytoplasm enriched fractions of individual strains were done in parallel.

### Sucrose Gradient Fractionation

Yeast strains were grown in galactose containing medium (YPG) at 30°C, centrifuged and resuspended in galactose (YPG) or glucose (YPD) containing medium. Cells were incubated at 30°C for two hours to a final OD of approximately 1.4. Ribosomes, preribosomes and polyribosomes were fractionated on sucrose gradients as described in [13] .

## Supporting Information

Figure S1 Polysome analyses of strains pGAL-RPL1, pGAL-RPL3 and pGAL-RPL40 after two hours shift to restrictive conditions. Polysome analyses of strains pGAL-RPL1 (TY933), pGAL-RPL3 (TY966) and pGAL-RPL40 (TY1104) were performed as described in Materials and Methods.
(2.33 MB TIF)

Figure S2 Strains used in this work. Strains used in this work with references or construction strategy are listed.
(0.07 MB DOC)

Figure S3 Plasmids used in this work. Plasmids used in this work with references or construction strategy are listed.
(0.06 MB DOC)

Figure S4 Oligonucleotides used in this work. Oligonucleotides used in this work are listed.
(0.07 MB DOC)

## References

[1] Zhang J, Harnpicharnchai P, Jakovljevic J, Tang L, Guo Y (2007). Assembly factors Rpf2 and Rrs1 recruit 5S rRNA and ribosomal proteins rpL5 and rpL11 into nascent ribosomes.. Genes Dev.

[2] Henras AK, Soudet J, Gérus M, Lebaron S, Caizergues-Ferrer M (2008). The post-transcriptional steps of eukaryotic ribosome biogenesis.. Cell Mol Life Sci.

[3] Liang X, Liu Q, Fournier MJ (2009). Loss of rRNA modifications in the decoding center of the ribosome impairs translation and strongly delays pre-rRNA processing.. http://www.ncbi.nlm.nih.gov/pubmed/19628622.

[4] Liang X, Liu Q, Fournier MJ (2007). rRNA modifications in an intersubunit bridge of the ribosome strongly affect both ribosome biogenesis and activity.. Mol Cell.

[5] Ferreira-Cerca S, Pöll G, Gleizes P, Tschochner H, Milkereit P (2005). Roles of eukaryotic ribosomal proteins in maturation and transport of pre-18S rRNA and ribosome function. Mol.. Cell.

[6] Moore PB (2009). The ribosome returned.. J Biol.

[7] Chandramouli P, Topf M, Ménétret J, Eswar N, Cannone JJ (2008). Structure of the mammalian 80S ribosome at 8.7 A resolution.. Structure.

[8] Spahn CMT, Gomez-Lorenzo MG, Grassucci RA, Jørgensen R, Andersen GR (2004). Domain movements of elongation factor eEF2 and the eukaryotic 80S ribosome facilitate tRNA translocation.. EMBO J.

[9] Ferreira-Cerca S, Pöll G, Kühn H, Neueder A, Jakob S (2007). Analysis of the in vivo assembly pathway of eukaryotic 40S ribosomal proteins.. Mol Cell.

[10] Ban N, Nissen P, Hansen J, Moore PB, Steitz TA (2000). The complete atomic structure of the large ribosomal subunit at 2.4 A resolution.. Science.

[11] Klein DJ, Moore PB, Steitz TA (2004). The roles of ribosomal proteins in the structure assembly, and evolution of the large ribosomal subunit.. J Mol Biol.

[12] Rosado IV, Kressler D, de la Cruz J (2007). Functional analysis of Saccharomyces cerevisiae ribosomal protein Rpl3p in ribosome synthesis.. Nucleic Acids Res.

[13] Deshmukh M, Tsay YF, Paulovich AG, Woolford JL (1993). Yeast ribosomal protein L1 is required for the stability of newly synthesized 5S rRNA and the assembly of 60S ribosomal subunits.. Mol Cell Biol.

[14] van Beekvelt CA, de Graaff-Vincent M, Faber AW, van't Riet J, Venema J (2001). All three functional domains of the large ribosomal subunit protein L25 are required for both early and late pre-rRNA processing steps in Saccharomyces cerevisiae.. Nucleic Acids Res.

[15] Martín-Marcos P, Hinnebusch AG, Tamame M (2007). Ribosomal protein L33 is required for ribosome biogenesis, subunit joining, and repression of GCN4 translation.. Mol Cell Biol.

[16] Moore JB, Farrar JE, Arceci RJ, Liu JM, Ellis SR (2009). Distinct ribosome maturation defects in yeast models of Diamond Blackfan anemia and Shwachman Diamond syndrome. Haematologica.. http://www.ncbi.nlm.nih.gov/pubmed/19713223.

[17] Robledo S, Idol RA, Crimmins DL, Ladenson JH, Mason PJ (2008). The role of human ribosomal proteins in the maturation of rRNA and ribosome production.. RNA.

[18] Gadal O, Strauss D, Kessl J, Trumpower B, Tollervey D (2001). Nuclear export of 60s ribosomal subunits depends on Xpo1p and requires a nuclear export sequence-containing factor, Nmd3p, that associates with the large subunit protein Rpl10p.. Mol Cell Biol.

[19] Hedges J, West M, Johnson AW (2005). Release of the export adapter, Nmd3p, from the 60S ribosomal subunit requires Rpl10p and the cytoplasmic GTPase Lsg1p.. EMBO J.

[20] Petitjean A, Bonneaud N, Lacroute F (1995). The duplicated Saccharomyces cerevisiae gene SSM1 encodes a eucaryotic homolog of the eubacterial and archaebacterial L1 ribosomal proteins.. Mol Cell Biol.

[21] Santos C, Ballesta JP (1994). Ribosomal protein P0, contrary to phosphoproteins P1 and P2, is required for ribosome activity and Saccharomyces cerevisiae viability.. J Biol Chem.

[22] Briones E, Briones C, Remacha M, Ballesta JP (1998). The GTPase center protein L12 is required for correct ribosomal stalk assembly but not for Saccharomyces cerevisiae viability.. J Biol Chem.

[23] Anderson SJ, Lauritsen JPH, Hartman MG, Foushee AMD, Lefebvre JM (2007). Ablation of ribosomal protein L22 selectively impairs alphabeta T cell development by activation of a p53-dependent checkpoint.. Immunity.

[24] Yu X, Warner JR (2001). Expression of a micro-protein.. J Biol Chem.

[25] Baronas-Lowell DM, Warner JR (1990). Ribosomal protein L30 is dispensable in the yeast Saccharomyces cerevisiae.. Mol Cell Biol.

[26] Peisker K, Braun D, Wölfle T, Hentschel J, Fünfschilling U (2008). Ribosome-associated complex binds to ribosomes in close proximity of Rpl31 at the exit of the polypeptide tunnel in yeast.. Mol Biol Cell.

[27] DeLabre ML, Kessl J, Karamanou S, Trumpower BL (2002). RPL29 codes for a non-essential protein of the 60S ribosomal subunit in Saccharomyces cerevisiae and exhibits synthetic lethality with mutations in genes for proteins required for subunit coupling.. Biochim Biophys Acta.

[28] Sachs AB, Davis RW (1990). Translation initiation and ribosomal biogenesis: involvement of a putative rRNA helicase and RPL46.. Science.

[29] Dresios J, Panopoulos P, Synetos D (2006). Eukaryotic ribosomal proteins lacking a eubacterial counterpart: important players in ribosomal function.. Mol Microbiol.

[30] Fried HM, Nam HG, Loechel S, Teem J (1985). Characterization of yeast strains with conditionally expressed variants of ribosomal protein genes tcm1 and cyh2.. Mol Cell Biol.

[31] Yon J, Giallongo A, Fried M (1991). The organization and expression of the Saccharomyces cerevisiae L4 ribosomal protein genes and their identification as the homologues of the mammalian ribosomal protein gene L7a.. Mol Gen Genet.

[32] Koller HT, Klade T, Ellinger A, Breitenbach M (1996). The yeast growth control gene GRC5 is highly homologous to the mammalian putative tumor suppressor gene QM.. Yeast.

[33] Donovan DM, Remington MP, Stewart DA, Crouse JC, Miles DJ (1990). Functional analysis of a duplicated linked pair of ribosomal protein genes in Saccharomyces cerevisiae.. Mol Cell Biol.

[34] Song JM, Cheung E, Rabinowitz JC (1996). Organization and characterization of the two yeast ribosomal protein YL19 genes.. Curr Genet.

[35] Rutgers CA, Schaap PJ, van 't Riet J, Woldringh CL, Raué HA (1990). In vivo and in vitro analysis of structure-function relationships in ribosomal protein L25 from Saccharomyces cerevisiae.. Biochim Biophys Acta.

[36] Finley D, Bartel B, Varshavsky A (1989). The tails of ubiquitin precursors are ribosomal proteins whose fusion to ubiquitin facilitates ribosome biogenesis.. Nature.

[37] Rivlin AA, Chan YL, Wool IG (1999). The contribution of a zinc finger motif to the function of yeast ribosomal protein YL37a.. J Mol Biol.

[38] Lucioli A, Presutti C, Ciafrè S, Caffarelli E, Fragapane P (1988). Gene dosage alteration of L2 ribosomal protein genes in Saccharomyces cerevisiae: effects on ribosome synthesis.. Mol Cell Biol.

[39] Ohtake Y, Wickner RB (1995). KRB1, a suppressor of mak7-1 (a mutant RPL4A), is RPL4B, a second ribosomal protein L4 gene, on a fragment of Saccharomyces chromosome XII.. Genetics.

[40] Hofer A, Bussiere C, Johnson AW (2007). Mutational Analysis of the Ribosomal Protein Rpl10 from Yeast.. J Biol Chem.

[41] Henry Y, Wood H, Morrissey JP, Petfalski E, Kearsey S (1994). The 5' end of yeast 5.8S rRNA is generated by exonucleases from an upstream cleavage site.. EMBO J.

[42] Faber AW, Vos HR, Vos JC, Raué HA (2006). 5'-end formation of yeast 5.8SL rRNA is an endonucleolytic event.. Biochem Biophys Res Commun.

[43] Farrar JE, Nater M, Caywood E, McDevitt MA, Kowalski J (2008). Abnormalities of the large ribosomal subunit protein, Rpl35a, in Diamond-Blackfan anemia.. Blood.

[44] Fatica A, Cronshaw AD, Dlakić M, Tollervey D (2002). Ssf1p prevents premature processing of an early pre-60S ribosomal particle.. Mol Cell.

[45] Briggs MW, Burkard KT, Butler JS (1998). Rrp6p, the yeast homologue of the human PM-Scl 100-kDa autoantigen, is essential for efficient 5.8 S rRNA 3' end formation.. J Biol Chem.

[46] van Hoof A, Lennertz P, Parker R (2000). Three conserved members of the RNase D family have unique and overlapping functions in the processing of 5S, 5.8S, U4, U5, RNase MRP and RNase P RNAs in yeast.. EMBO J.

[47] Faber AW, Van Dijk M, Raué HA, Vos JC (2002). Ngl2p is a Ccr4p-like RNA nuclease essential for the final step in 3'-end processing of 5.8S rRNA in Saccharomyces cerevisiae.. RNA.

[48] Gabel HW, Ruvkun G (2008). The exonuclease ERI-1 has a conserved dual role in 5.8S rRNA processing and RNAi.. Nat Struct Mol Biol.

[49] Geerlings TH, Vos JC, Raué HA (2000). The final step in the formation of 25S rRNA in Saccharomyces cerevisiae is performed by 5'-->3' exonucleases.. RNA.

[50] Trapman J, Planta RJ (1976). Maturation of ribosomes in yeast. I Kinetic analysis by labelling of high molecular weight rRNA species.. Biochim Biophys Acta.

[51] Trapman J, Planta RJ, Raué HA (1976). Maturation of ribosomes in yeast. II. Position of the low molecular weight rRNA species in the maturation process.. Biochim Biophys Acta.

[52] Smitt WW, Vlak JM, Schiphof R, Rozijn TH (1972). Precursors of ribosomal RNA in yeast nucleus. Biosynthesis and relation to cytoplasmic ribosomal RNA.. Exp Cell Res.

[53] Saveanu C, Namane A, Gleizes P, Lebreton A, Rousselle J (2003). Sequential protein association with nascent 60S ribosomal particles.. Mol Cell Biol.

[54] Yeh LC, Lee JC (1990). Structural analysis of the internal transcribed spacer 2 of the precursor ribosomal RNA from Saccharomyces cerevisiae.. J Mol Biol.

[55] Joseph N, Krauskopf E, Vera MI, Michot B (1999). Ribosomal internal transcribed spacer 2 (ITS2) exhibits a common core of secondary structure in vertebrates and yeast.. Nucleic Acids Res.

[56] Côté CA, Greer CL, Peculis BA (2002). Dynamic conformational model for the role of ITS2 in pre-rRNA processing in yeast.. RNA.

[57] Zengel JM, Jerauld A, Walker A, Wahl MC, Lindahl L (2003). The extended loops of ribosomal proteins L4 and L22 are not required for ribosome assembly or L4-mediated autogenous control.. RNA.

[58] Timsit Y, Acosta Z, Allemand F, Chiaruttini C, Springer M (2009). The Role of Disordered Ribosomal Protein Extensions in the Early Steps of Eubacterial 50 S Ribosomal Subunit Assembly.. Int J Mol Sci.

[59] Röhl R, Nierhaus KH (1982). Assembly map of the large subunit (50S) of Escherichia coli ribosomes.. Proc Natl Acad Sci USA.

[60] Shuai K, Warner JR (1991). A temperature sensitive mutant of Saccharomyces cerevisiae defective in pre-rRNA processing.. Nucleic Acids Res.

[61] Lindahl L, Archer RH, Zengel JM (1992). A new rRNA processing mutant of Saccharomyces cerevisiae.. Nucleic Acids Res.

[62] Kufel J, Dichtl B, Tollervey D (1999). Yeast Rnt1p is required for cleavage of the pre-ribosomal RNA in the 3' ETS but not the 5' ETS.. RNA.

[63] Allmang C, Kufel J, Chanfreau G, Mitchell P, Petfalski E (1999). Functions of the exosome in rRNA, snoRNA and snRNA synthesis.. EMBO J.

[64] Schäfer T, Maco B, Petfalski E, Tollervey D, Böttcher B (2006). Hrr25-dependent phosphorylation state regulates organization of the pre-40S subunit.. Nature.

[65] Baudin-Baillieu A, Tollervey D, Cullin C, Lacroute F (1997). Functional analysis of Rrp7p, an essential yeast protein involved in pre-rRNA processing and ribosome assembly.. Mol Cell Biol.

[66] Iouk TL, Aitchison JD, Maguire S, Wozniak RW (2001). Rrb1p, a yeast nuclear WD-repeat protein involved in the regulation of ribosome biosynthesis.. Mol Cell Biol.

[67] West M, Hedges JB, Chen A, Johnson AW (2005). Defining the order in which Nmd3p and Rpl10p load onto nascent 60S ribosomal subunits.. Mol Cell Biol.

[68] Burke D, Dawson D, Stearns T (2000). Methods in Yeast Genetics, 2000 Edition : A Cold Spring Harbor Laboratory Course Manual. 1. ed..

[69] Longtine MS, McKenzie A, Demarini DJ, Shah NG, Wach A (1998). Additional modules for versatile and economical PCR-based gene deletion and modification in Saccharomyces cerevisiae.. Yeast.

[70] Milkereit P, Strauss D, Bassler J, Gadal O, Kühn H (2003). A Noc complex specifically involved in the formation and nuclear export of ribosomal 40 S subunits.. J Biol Chem.

[71] Venema J, Planta RJ, Raué HA (1998). In vivo mutational analysis of ribosomal RNA in Saccharomyces cerevisiae.. Methods Mol Biol.

[72] Sambrook J, Fritsch E, Maniatis T (1989). Molecular Cloning: A Laboratory Manual. 2undefined ed..

