# Supplementary figures and images for: rRNA Maturation in Yeast Cells Depleted of Large Ribosomal Subunit Proteins
Source: PLoS One. 2009 Dec 11;4(12):e8249. doi: 10.1371/journal.pone.0008249 (PMC2788216; doi:10.1371/journal.pone.0008249)

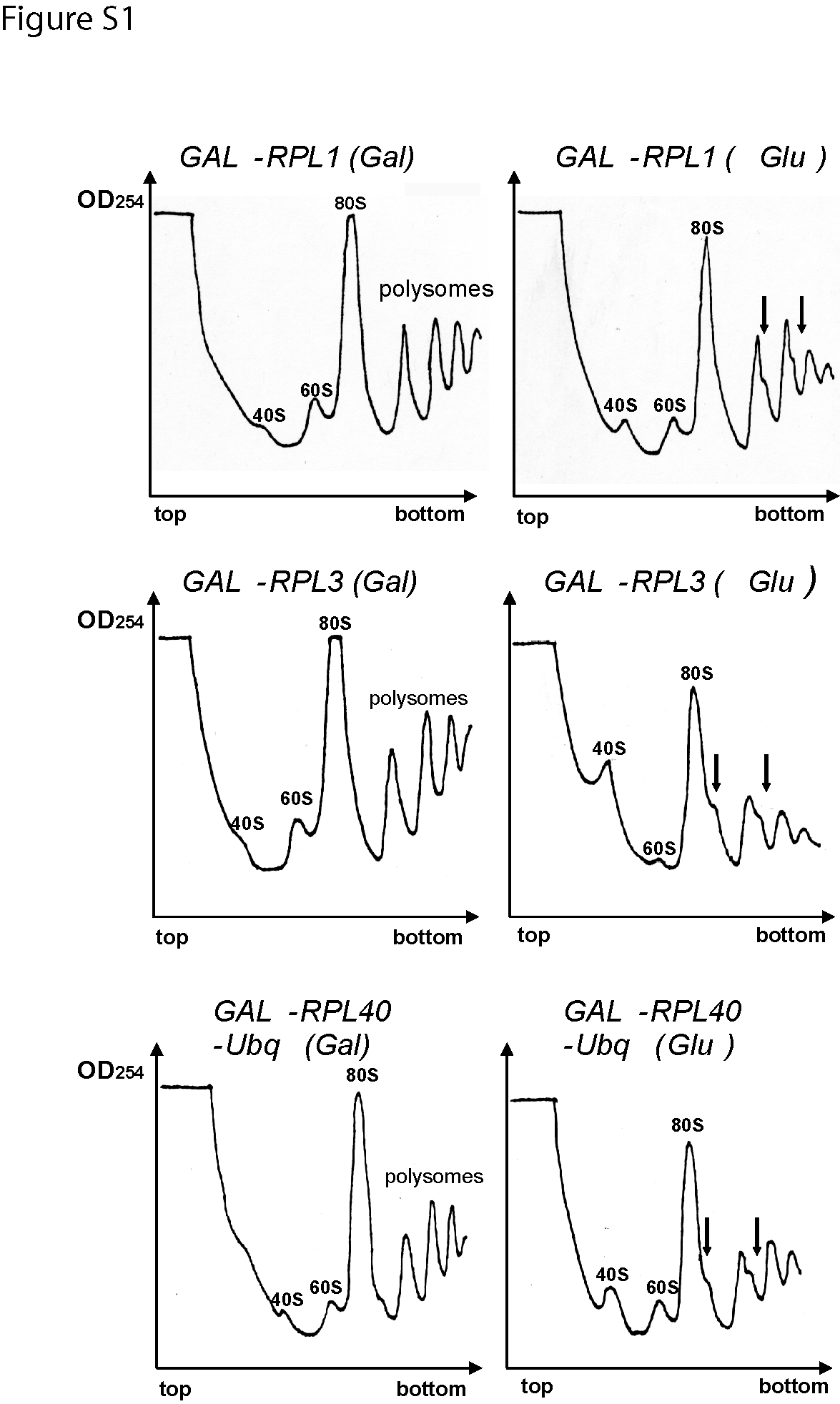

Supplement: Figure S1 — Polysome analyses of strains pGAL-RPL1, pGAL-RPL3 and pGAL-RPL40 after two hours shift to restrictive conditions. Polysome analyses of strains pGAL-RPL1 (TY933), pGAL-RPL3 (TY966) and pGAL-RPL40 (TY1104) were performed as described in Materials and Methods. (2.33 MB TIF) [file pone.0008249.s001.tif]
