# Supplementary material for: rRNA Maturation in Yeast Cells Depleted of Large Ribosomal Subunit Proteins
Source: PLoS One. 2009 Dec 11;4(12):e8249. doi: 10.1371/journal.pone.0008249 (PMC2788216; doi:10.1371/journal.pone.0008249)
Supplement: Figure S2 — Strains used in this work. Strains used in this work with references or construction strategy are listed. (0.07 MB DOC) [file pone.0008249.s002.doc]

**Figure S2**

| **Strain** | **Name** | **Plasmid** | **Genotype** | **Construction** |  |
| --- | --- | --- | --- | --- | --- |
| **TY927** | pGAL-RPL10 | TK808 | his3-1/leu2-0/ura3-0/YLR075w::KANMX4 | Strain Y22686 (Euroscarf) was transformed with plasmid TK795 (RPL10/URA3) and tetrade analyses was performed. 5-FoA sensitive, uracile prototrophe and geniticine resistant offsprings were selected and transformed with plasmid TK808 (pGAL1-RPL10/LEU2). Selection for loss of plasmid TK795 was done on 5-FoA containing plates and the resulting strain showed galactose dependent growth. | |
| **TY928** | pGAL-RPL23 | TK812 | his3-1/leu2-0/ura3-0/YBL087c::HIS3MX6/YER117w::KANMX4 | The KANMX4 cassette in strain Y03113 (Euroscarf) was replaced by the HIS3MX6 cassette from plasmid pFA6a-GFP(S65T)-HIS3MX6 (Longtine). The resulting strain was crossed with Y16115 (Euroscarf) and transformed with plasmid TK800 (RPL23B/URA3). Tetrade analyses was performed and 5-Fluorouritidylc acid sensitiv (5-FoA) , histidine and uracile prototrophe and geniticine resistant offsprings were selected and transformed with plasmid TK812 (pGAL1-RPL23B/LEU2). Selection for loss of plasmid TK800 was done on 5-FoA containing plates and the resulting strain showed galactose dependent growth. | |
| **TY930** | pGAL-RPL34 | TK814 | his3-1/leu2-0/ura3-0/YIL052c::HIS3MX6/YER056c-a::KANMX4 | The KANMX4 cassette in strain Y11445 (Euroscarf) was replaced by the HIS3MX6 cassette (see above). The resulting strain was crossed with Y00192 (Euroscarf) and transformed with plasmid TK802 (RPL34B/URA3). Tetrade analyses was performed and 5-FoA sensitive , histidine and uracile prototrophe and geniticine resistant offsprings were selected and transformed with plasmid TK814 (pGAL1-RPL34B/LEU2). Selection for loss of plasmid TK802 was done on 5-FoA containing plates and the resulting strain showed galactose dependent growth. | |
| **TY931** | pGAL-RPL16 | TK810 | his3-1/leu2-0/ura3-0/YNL069c::KANMX4/YIL133c::HIS3MX6 | The KANMX4 cassette in strain Y02292 (Euroscarf) was replaced by the HIS3MX6 cassette (see above). The resulting strain was crossed with Y17376 (Euroscarf) and transformed with plasmid TK797 (RPL16B/URA3). Tetrade analyses was performed and 5-FoA sensitive , histidine and uracile prototrophe and geniticine resistant offsprings were selected and transformed with plasmid TK810 (pGAL1-RPL16B/LEU2). Selection for loss of plasmid TK797 was done on 5-FoA containing plates and the resulting strain showed galactose dependent growth. | |
| **TY932** | pGAL-RPL20 | TK821 | his3-1/leu2-0/ura3-0/YOR312c::HIS3/YMR242c::KANMX4 | The KANMX4 cassette in strainY11609 (Euroscarf) was replaced by the HIS3MX6 cassette (see above). The resulting strain was crossed with Y00828 (Euroscarf) and transformed with plasmid TK799 (RPL20B/URA3). Tetrade analyses was performed and 5-FoA sensitive , histidine and uracile prototrophe and geniticine resistant offsprings were selected and transformed with plasmid TK821 (pGAL1-RPL20B/LEU2). Selection for loss of plasmid TK799 was done on 5-FoA containing plates and the resulting strain showed galactose dependent growth | |
| **TY933** | pGAL-RPL1 | TK818 | his3-1/leu2-0/ura3-0/YGL135W::KANMX4/YPL220w::HIS3MX6 | The KANMX4 cassette in strainY01072 (Euroscarf) was replaced by the HIS3MX6 cassette (see above). The resulting strain was crossed with Y14502 (Euroscarf) and transformed with plasmid TK815 (RPL1B/URA3). Tetrade analyses was performed and 5-FoA sensitive , histidine and uracile prototrophe and geniticine resistant offsprings were selected and transformed with plasmid TK818 (pGAL1-RPL1B/LEU2). Selection for loss of plasmid TK815 was done on 5-FoA containing plates and the resulting strain showed galactose dependent growth | |
| **TY965** | pGAL-RPL2 | TK819 | his3-1/leu2-0/ura3-0/YIL018w::KANMX4/YFR031c-a::HIS3MX6 | The KANMX4 cassette in strainY05712 (Euroscarf) was replaced by the HIS3MX6 cassette (see above). The resulting strain was crossed with Y11411 (Euroscarf) and transformed with plasmid TK847 (RPL2B/URA3). Tetrade analyses was performed and 5-FoA sensitive , histidine and uracile prototrophe and geniticine resistant offsprings were selected and transformed with plasmid TK819 (pGAL1-RPL2B/LEU2). Selection for loss of plasmid TK847 was done on 5-FoA containing plates and the resulting strain showed galactose dependent growth | |
| **TY966** | pGAL-RPL3 | TK822 | his3-1/leu2-0/ura3-0/YOR063w::KANMX4 | Strain Y21839 (Euroscarf) was transformed with plasmid TK816 (RPL3/URA3) and tetrade analyses was performed. 5-FoA sensitive, uracile prototrophe and geniticine resistant offsprings were selected and transformed with plasmid TK966 (pGAL1-RPL3/LEU2). Selection for loss of plasmid TK816 was done on 5-FoA containing plates and the resulting strain showed galactose dependent growth. | |
| **TY967** | pGAL-RPL13 | TK823 | his3-1/leu2-0/ura3-0/YMR142c::KANMX4/YDL082w::HIS3MX6 | The KANMX4 cassette in strainY03779 (Euroscarf) was replaced by the HIS3MX6 cassette (see above). The resulting strain was crossed with Y16443 (Euroscarf) and transformed with plasmid TK796 (RPL13A/URA3). Tetrade analyses was performed and 5-FoA sensitive , histidine and uracile prototrophe and geniticine resistant offsprings were selected and transformed with plasmid TK823 (pGAL1-RPL13A/LEU2). Selection for loss of plasmid TK796 was done on 5-FoA containing plates and the resulting strain showed galactose dependent growth | |
| **TY1026** | pGAL-RPL25 | TK865 | his3-1/leu2-0/ura3-0/YOL127w::KANMX4 | Strain Y26277 (Euroscarf) was cultivated in galactose containing medium, transformed with plasmid TK865 (pGAL1-RPL25/LEU2) and tetrade analyses was performed. 5-FoA sensitive, leucine prototrophe and geniticine resistant offsprings were selected. The resulting strain showed galactose dependent growth. | |
| **TY1082** | pGal-RPL27 | TK855 | his3-1/leu2-0/ura3-0/YDR471w::HIS3MX6/YHR010w::KANMX4 | Strain Y30973 (Euroscarf) was cultivated in galactose containing media and transformed with plasmid TK855 (pGAL1-RPL27A/LEU2). Tetrade analysis was performed and a geniticine resistant and leucine prototroph offspring was crossed with Y14305 (Euroscarf) in which the KANMX4 cassette was replaced by the HIS3MX6 cassette (see above). Tetrade analyses was performed and histidine and leucine prototrophe and geniticine resistant offsprings were selected. The resulting strain showed galactose dependent growth | |
| **TY1092** | pGAL-RPL5 | TK852 | his3-1/leu2-0/ura3-0/YPL131w::KANMX4 | Strain Y26478 (Euroscarf) was transformed with plasmid TK793 (RPL5/URA3) and tetrade analyses was performed. 5-FoA sensitive, uracile prototrophe and geniticine resistant offsprings were selected and transformed with plasmid TK852 (pGAL1-RPL5/LEU2). Selection for loss of plasmid TK793 was done on 5-FoA containing plates and the resulting strain showed galactose dependent growth. | |
| **TY1093** | pGAL-RPL28 | TK856 | his3-1/leu2-0/ura3-0/YGL103w::KANMX4 | Strain Y24470 (Euroscarf) was transformed with plasmid TK848 (RPL28/URA3) and tetrade analyses was performed. 5-FoA sensitive, uracile prototrophe and geniticine resistant offsprings were selected and transformed with plasmid TK856 (pGAL1-RPL28/LEU2). Selection for loss of plasmid TK848 was done on 5-FoA containing plates and the resulting strain showed galactose dependent growth. | |
| **TY1094** | pGAL-RPL35 | TK857 | his3-1/leu2-0/ura3-0/YDL191w::KANMX4/YDL136w::HIS3MX6 | The KANMX4 cassette in strainY13834 (Euroscarf) was replaced by the HIS3MX6 cassette (see above). The resulting strain was crossed with strain Y13834 (Euroscarf) and transformed with plasmid TK851 (RPL35A/URA3). Tetrade analyses was performed in which increased formation of triads and low tetrade formation was observed, and 5-FoA sensitive , histidine and uracile prototrophe and geniticine resistant offsprings were selected and transformed with plasmid TK857 (pGAL1-RPL35A/LEU2). Selection for loss of plasmid TK851 was done on 5-FoA containing plates and the resulting strain showed galactose dependent growth | |
| **TY1095** | pGAL-RPL17 | TK846 | his3-1/leu2-0/ura3-0/YKL180w::KANMX4/YJL177w::HIS3MX6 | Strain Y25030 (Euroscarf) was transformed with plasmid TK798 (RPL17A/URA3) and tetrade analyses was performed. 5-FoA sensitive, uracile prototrophe and geniticine resistant offsprings were selected and transformed with plasmid K846 (pGAL1-RPL17A/LEU2). Selection for loss of plasmid TK798 was done on 5-FoA containing plates and the resulting strain showed slow growth on glucose containing medium. It was subsequently transformed with a PCR product containing the HIS3KANMX6 cassette flanked by promoter and terminator regions of RPL17B. Histidine prototroph and geneticine resistant clones were selected. The resulting strain showed galactose dependent growth | |
| **TY1096** | pGAL-RPL33 | TK813 | his3-1/leu2-0/ura3-0/YPL143w::KANMX4/YOR234c::HIS3MX6 | Strain Y22109 (Euroscarf) was transformed with plasmid TK801 (RPL33A/URA3) and tetrade analyses was performed. 5-FoA sensitive, uracile prototrophe and geniticine resistant offsprings were selected and transformed with plasmid TK813 (pGAL1-RPL33A/LEU2). Selection for loss of plasmid TK801 was done on 5-FoA containing plates and the resulting strain showed slow growth on glucose containing medium. It was subsequently transformed with a PCR product containing the HIS3KANMX6 cassette flanked by promoter and terminator regions of RPL33B. Histidine prototroph and geneticine resistant clones were selected. The resulting strain showed galactose dependent growth | |
| **TY1097** | pGAL-RPL8 | TK882 | his3-1/leu2-0/ura3-0/YHL033c::HIS3MX6/YLL045c::KANMX4 | The KANMX4 cassette in strainY00930 (Euroscarf) was replaced by the HIS3MX6 cassette (see above). The resulting strain was crossed with Y11533 (Euroscarf) and transformed with plasmid TK889 (RPL8B/URA3). Tetrade analyses was performed and 5-FoA sensitive , histidine and uracile prototrophe and geniticine resistant offsprings were selected and transformed with plasmid TK882 (pGAL1-RPL8B/LEU2). Selection for loss of plasmid TK889 was done on 5-FoA containing plates and the resulting strain showed galactose dependent growth | |
| **TY1098** | pGAL-RPL9 | TK883 | his3-1/leu2-0/ura3-0/YNL067w::KANMX4/YGL147c::HIS3MX6 | The KANMX4 cassette in strainY04514 (Euroscarf) was replaced by the HIS3MX6 cassette (see above). The resulting strain was crossed with Y17215 (Euroscarf) and transformed with plasmid TK890 (RPL9A/URA3). Tetrade analyses was performed and 5-FoA sensitive , histidine and uracile prototrophe and geniticine resistant offsprings were selected and transformed with plasmid TK883 (pGAL1-RPL9A/LEU2). Selection for loss of plasmid TK890 was done on 5-FoA containing plates and the resulting strain showed galactose dependent growth | |
| **TY1099** | pGAL-RPL19 | TK884 | his3-1/leu2-0/ura3-0/YBR084c-a::HIS3MX6/YBL027w::KANMX4 | The KANMX4 cassette in strainY07156 (Euroscarf) was replaced by the HIS3MX6 cassette (see above). The resulting strain was crossed with Y13053 (Euroscarf) and transformed with plasmid TK891 (RPL19B/URA3). Tetrade analyses was performed and 5-FoA sensitive , histidine and uracile prototrophe and geniticine resistant offsprings were selected and transformed with plasmid TK884 (pGAL1-RPL19B/LEU2). Selection for loss of plasmid TK891 was done on 5-FoA containing plates and the resulting strain showed galactose dependent growth | |
| **TY1100** | pGAL-RPL21 | TK885 | his3-1/leu2-0/ura3-0/YBR191w::HIS3MX6/YPL079w::KANMX4 | The KANMX4 cassette in strainY06679 (Euroscarf) was replaced by the HIS3MX6 cassette (see above). The resulting strain was crossed with Y12749 (Euroscarf) and transformed with plasmid TK892 (RPL21A/URA3). Tetrade analyses was performed and 5-FoA sensitive , histidine and uracile prototrophe and geniticine resistant offsprings were selected and transformed with plasmid TK885 (pGAL1-RPL21A/LEU2). Selection for loss of plasmid TK892 was done on 5-FoA containing plates and the resulting strain showed galactose dependent growth | |
| **TY1102** | pGAL-RPL32 | TK880 | his3-1/leu2-0/ura3-0/YBL092w::KANMX4 | Strain Y23118 (Euroscarf) was transformed with plasmid TK894 (RPL32/URA3) and tetrade analyses was performed. 5-FoA sensitive, uracile prototrophe and geniticine resistant offsprings were selected and transformed with plasmid TK880 (pGAL1-RPL32/LEU2). Selection for loss of plasmid TK894 was done on 5-FoA containing plates and the resulting strain showed galactose dependent growth. | |
| **TY1103** | pGAL-RPL43 | TK881 | his3-1/leu2-0/ura3-0/YJR094w-a::HIS3MX6/YPR043w::KANMX4 | The KANMX4 cassette in strainY16906 (Euroscarf) was replaced by the HIS3MX6 cassette (see above). The resulting strain was crossed with Y07148 (Euroscarf) and transformed with plasmid TK896 (RPL43A/URA3). Tetrade analyses was performed and 5-FoA sensitive , histidine and uracile prototrophe and geniticine resistant offsprings were selected and transformed with plasmid TK881 (pGAL1-RPL43A/LEU2). Selection for loss of plasmid TK896 was done on 5-FoA containing plates and the resulting strain showed galactose dependent growth | |
| **TY1104** | pGAL-RPL40 | TK888 | his3-1/leu2-0/ura3-0/YIL148w::HIS3MX6/YKR094c::KANMX4 | The KANMX4 cassette in strainY02307 (Euroscarf) was replaced by the HIS3MX6 cassette (see above). The resulting strain was crossed with Y17103 (Euroscarf) and transformed with plasmid TK895 (RPL40A/URA3). Tetrade analyses was performed and 5-FoA sensitive , histidine and uracile prototrophe and geniticine resistant offsprings were selected and transformed with plasmid TK888 (pGAL1-RPL40/LEU2). Selection for loss of plasmid TK895 was done on 5-FoA containing plates and the resulting strain showed galactose dependent growth | |
| **JW8402** | pGAL-RPL4 | no plasmid | ura3-52/trp1-101/lys2-801/his3-d200/leu2-d1/rpl4b::KANMX6/GAL-3HA-RPL4A (TRP1) | Strains JWY8402 was generated as described in Longtine et al (1998). One chromosomal copy of each gene was deleted and another copy was placed under the control of galactose-inducible promoter. | |
| **JW8423** | pGAL-RPL7 | no plasmid | ura3-52/trp1-101/lys2-801/his3-d200/leu2-d1/rpl7b::KANMX6/GAL-3HA-RPL7A (TRP1) | Strain JWY8423 was generated as described in Longtine et al (1998). One chromosomal copy of each gene was deleted and another copy was placed under the control of galactose-inducible promoter. | |
| **JW8425** | pGAL-RPL18 | no plasmid | ura3-52/trp1-101/lys2-801/his3-d200/leu2-d1/rpl18b::KANMX6/GAL-3HA-RPL18A (TRP1) | Strain JWY8425 was generated as described in Longtine et al (1998). One chromosomal copy of each gene was deleted and another copy was placed under the control of galactose-inducible promoter. | |
