# Supplementary material for: rRNA Maturation in Yeast Cells Depleted of Large Ribosomal Subunit Proteins
Source: PLoS One. 2009 Dec 11;4(12):e8249. doi: 10.1371/journal.pone.0008249 (PMC2788216; doi:10.1371/journal.pone.0008249)
Supplement: Figure S3 — Plasmids used in this work. Plasmids used in this work with references or construction strategy are listed. (0.06 MB DOC) [file pone.0008249.s003.doc]

**Figure S3**

| **Vector** | **Cloning strategy** |
| --- | --- |
| **Ycplac33** | Gietz and Sugino, Gene 74 (2), p. 527-534 |
| **TTK230** | Ferreira-Cerca et al., Mol. Cell 20 (2), p. 263-275 |
| **TK793** | PCR product of a PCR reaction with yeast genomic DNA as template and primers O1224/O1225 was cloned BamHI/SalI in Ycplac33 |
| **TK795** | PCR product of a PCR reaction with yeast genomic DNA as template and primers O1230/O1231 was cloned BamHI/PstI inYcplac33 |
| **TK796** | PCR product of a PCR reaction with yeast genomic DNA as template and primers O1232/O1233 was cloned BamHI/PstI in Ycplac33 |
| **TK797** | PCR product of a PCR reaction with yeast genomic DNA as template and primers O1234/O1235 was cloned BamHI/PstI in Ycplac33 |
| **TK798** | PCR product of a PCR reaction with yeast genomic DNA as template and primers O1236/O1237 was cloned BamHI/PstI in Ycplac33 |
| **TK799** | PCR product of a PCR reaction with yeast genomic DNA as template and primers O1238/O1239 was cloned BamHI/PstI in Ycplac33 |
| **TK800** | PCR product of a PCR reaction with yeast genomic DNA as template and primers O1242/O1243 was cloned BamHI/PstI in Ycplac33 |
| **TK801** | PCR product of a PCR reaction with yeast genomic DNA as template and primers O1252/O1253 was cloned BamHI/PstI in Ycplac33 |
| **TK802** | PCR product of a PCR reaction with yeast genomic DNA as template and primers O1254/O1255 was cloned BamHI/PstI in Ycplac33 |
| **TK808** | PCR product of a PCR reaction with yeast genomic DNA as template and primers O1279/O1231 was cloned BamHI/PstI in TK230, sequence was confirmed |
| **TK810** | PCR product of a PCR reaction with yeast genomic DNA as template and primers O1281/O1235 was cloned BamHI/PstI in TK230, sequence was confirmed |
| **TK812** | PCR product of a PCR reaction with yeast genomic DNA as template and primers O1284/O1297 was cloned BamHI/PstI in TK230, sequence was confirmed |
| **TK813** | PCR product of a PCR reaction with yeast genomic DNA as template and primers O1289/O1253 was cloned BamHI/PstI in TK230, sequence was confirmed |
| **TK814** | PCR product of a PCR reaction with yeast cDNA as template and primers O1290/O1299 was cloned BamHI/PstI in TK230 sequence was confirmed |
| **TK815** | PCR product of a PCR reaction with yeast genomic DNA as template and primers O1216/O1217 was cloned BamHI/PstI in Ycplac33 |
| **TK816** | PCR product of a PCR reaction with yeast genomic DNA as template and primers O1220/O1221 was cloned BamHI/PstI in Ycplac33 |
| **TK818** | PCR product of a PCR reaction with yeast genomic DNA as template and primers O1272/O1217 was cloned BamHI/PstI in TK230 sequence was confirmed |
| **TK819** | PCR product of a PCR reaction with yeast genomic DNA as template and primers O1273/O1219 was cloned BamHI/PstI in TK230 sequence was confirmed |
| **TK821** | PCR product of a PCR reaction with yeast genomic DNA as template and primers O1282/O1239 was cloned BamHI/PstI in TK230 sequence was confirmed |
| **TK822** | PCR product of a PCR reaction with yeast genomic DNA as template and primers O1274/O1221 was cloned BamHI/PstI in TK230 sequence was confirmed |
| **TK823** | PCR product of a PCR reaction with yeast genomic DNA as template and primers O1280/O1233 was cloned BamHI/PstI in TK230, sequence was confirmed |
| **TK846** | PCR product of a PCR reaction with yeast cDNA as template and primers O1300/O1302 was cloned BamHI/PstI in TK230, sequence was confirmed |
| **TK847** | PCR product of a PCR reaction with yeast genomic DNA as template and primers O1218/O1219 was cloned BamHI/PstI in Ycplac33 |
| **TK848** | PCR product of a PCR reaction with yeast genomic DNA as template and primers O1384/O1385 was cloned BamHI/PstI in Ycplac33 |
| **TK851** | PCR product of a PCR reaction with yeast genomic DNA as template and primers O1256/O1257 was cloned BamHI/SalI in Ycplac33 |
| **TK852** | PCR product of a PCR reaction with yeast cDNA as template and primers O1276/O1225 was cloned BamHI/PstI in TK230, sequence was confirmed |
| **TK855** | PCR product of a PCR reaction with yeast cDNA as template and primers O1287/O1294 was cloned BamHI/PstI in TK230 sequence was confirmed |
| **TK856** | PCR product of a PCR reaction with yeast cDNA as template and primers O1288/O1298 was cloned BamHI/PstI in TK230 sequence was confirmed |
| **TK857** | PCR product of a PCR reaction with yeast cDNA as template and primers O1291/O1295 was cloned BamHI/PstI in TK230 sequence was confirmed |
| **TK865** | PCR product of a PCR reaction with yeast genomic DNA as template and primers O1285/O1245 was cloned BamHI/PstI in TK230 |
| **TK880** | PCR product of a PCR reaction with yeast genomic DNA as template and primers O1436/O1405 was cloned BamHI/PstI in TK230, sequence was confirmed |
| **TK881** | PCR product of a PCR reaction with yeast genomic DNA as template and primers O1442/O1443 was cloned BamHI/PstI in TK230, sequence was confirmed |
| **TK882** | PCR product of a PCR reaction with yeast genomic DNA as template and primers O1429/O1387 was cloned BamHI/PstI in TK230, has on position 179 exchange of Isoleucine to valine |
| **TK883** | PCR product of a PCR reaction with yeast genomic DNA as template and primers O1430/1389 was cloned BamHI/PstI in TK230, sequence was confirmed |
| **TK884** | PCR product of a PCR reaction with yeast genomic DNA as template and primers O1432/1397 was cloned BamHI/PstI in TK230, sequence was confirmed |
| **TK885** | PCR product of a PCR reaction with yeast genomic DNA as template and primers O1433/1399 was cloned BamHI/PstI in TK230, sequence was confirmed |
| **TK888** | PCR product of a PCR reaction with yeast genomic DNA as template and primers O1439/O1424 was cloned BamHI/PstI in TK230, sequence was confirmed, lacks N-terminal ubiquitine moiety |
| **TK889** | PCR product of a PCR reaction with yeast genomic DNA as template and primers O1386/O1387 was cloned BamHI/PstI in Ycplac33 |
| **TK890** | PCR product of a PCR reaction with yeast genomic DNA as template and primers O1388/O1389 was cloned BamHI/PstI in Ycplac33 |
| **TK891** | PCR product of a PCR reaction with yeast genomic DNA as template and primers O1396/O1397 was cloned BamHI/PstI in Ycplac33 |
| **TK892** | PCR product of a PCR reaction with yeast genomic DNA as template and primers O1398/O1399 was cloned BamHI/PstI in Ycplac33 |
| **TK894** | PCR product of a PCR reaction with yeast genomic DNA as template and primers O1404/O1405 was cloned BamHI/PstI in Ycplac33 |
| **TK895** | PCR product of a PCR reaction with yeast genomic DNA as template and primers O1412/O1413 was cloned BamHI/PstI in Ycplac33 |
| **TK896** | PCR product of a PCR reaction with yeast genomic DNA as template and primers O1441/O1443 was cloned BamHI/PstI in Ycplac33 |
