# Supplementary material for: rRNA Maturation in Yeast Cells Depleted of Large Ribosomal Subunit Proteins
Source: PLoS One. 2009 Dec 11;4(12):e8249. doi: 10.1371/journal.pone.0008249 (PMC2788216; doi:10.1371/journal.pone.0008249)
Supplement: Figure S4 — Oligonucleotides used in this work. Oligonucleotides used in this work are listed. (0.07 MB DOC) [file pone.0008249.s004.doc]

**Figure S4**

| **Oligo** | **Sequence** |
| --- | --- |
| **O1216** | 5'-TTTTTTGGATCCGAATACGGACATTTTTGAACC-3' |
| **O1217** | 5'-TTTTTTCTGCAGATGGTTGAACTGATTGAACAC-3' |
| **O1218** | 5'-TTTTTTGGATCCCAAGTTTTCAGACTAGAACCC-3' |
| **O1219** | 5'-TTTTTTCTGCAGTACTATATCACATGGACCTGC-3' |
| **O1220** | 5'-TTTTTTGGATCCCGGACAGTAATATAGTAATCG-3' |
| **O1221** | 5'-TTTTTTCTGCAGATTGTAGCAAAGATTGTAAGG-3' |
| **O1224** | 5'-TTTTTTGGATCCTAGACAGCTAACTTGTACATC-3' |
| **O1225** | 5'-TTTTTTGTCGACAAATCTTCATATGTTCGTTAC-3' |
| **O1230** | 5'-TTTTTTGGATCCTAGTAGCGGTTATTTCCGTGG-3' |
| **O1231** | 5'-CTCGTGAATAATCTGAATGAC-3' |
| **O1232** | 5'-TTTTTTGGATCCCACAATGCATTATTTTTCAAC-3' |
| **O1233** | 5'-TTTTTTCTGCAGTTGTTCAATACCATCTTTCGC-3' |
| **O1234** | 5'-TTTTTTGGATCCTTCAATCCGTACACTATACAC-3' |
| **O1235** | 5'-TTTTTTCTGCAGGAATTACTTGACCGTTACTGC-3' |
| **O1236** | 5'-TTTTTTGGATCCAACTCAAGACTATTATCATCC-3' |
| **O1237** | 5'-TTTTTTCTGCAGATGAAGAACGCACACGTTCCG-3' |
| **O1238** | 5'-TTTTTTGGATCCTTCGGGACTGTTCACCCGTAC-3' |
| **O1239** | 5'-TTTTTTCTGCAGAATCCAAATGACAGAAATGCG-3' |
| **O1242** | 5'-TTTTTTGGATCCTAATATCTGACATATTATCCC-3' |
| **O1243** | 5'-TTTTTTCTGCAGAATGATTAACAATCTTATAGC-3' |
| **O1245** | 5'-TTTTTTCTGCAGGAAACAGCGGCGTTGCTGATG-3' |
| **O1252** | 5'-TTTTTTGGATCCGGCAATATTAACATCCTTACC-3' |
| **O1253** | 5'-TTTTTTCTGCAGTCAATCATAGCTTCACTCCGC-3' |
| **O1254** | 5'-TTTTTTGGATCCCAACTTTGCGGTGTACAGATG-3' |
| **O1255** | 5'-TTTTTTCTGCAGCCCTCCATTTCCGACCAATTG-3' |
| **O1256** | 5'-TTTTTTGGATCCAATCTGGGGACTGTATTAATC-3' |
| **O1257** | 5'-TTTTTTGTCGACTGACCCGTTTAATAGAATGCC-3' |
| **O1272** | 5'-TTTTTTGGATCCATGTCTAAGATCACCTCTTCT-3' |
| **O1273** | 5'-TTTTTTGGATCCATGGGTAGAGTTATTCGTAACCAA-3' |
| **O1274** | 5'-TTTTTTGGATCCATGTCTCACAGAAAGTACGAAG-3' |
| **O1276** | 5'-TTTTTTGGATCCATGGCTTTCCAAAAAGACGC-3' |
| **O1279** | 5'-TTTTTTGGATCCATGGCTAGAAGACCAGCTAG-3' |
| **O1280** | 5'-TTTTTTGGATCCATGGCCATTTCCAAGAATTTACCA-3' |
| **O1281** | 5'-TTTTTTGGATCCATGTCTCAACCAGTCGTTGTTATTGATGCTAAGGATCATTTGTTGGGTCG-3' |
| **O1282** | 5'-TTTTTTGGATCCATGTATTTGGCTCATTTCAAAGAATAC-3' |
| **O1284** | 5'-TTTTTTGGATCCATGTCAGGTAACGGTGCTCAAG-3' |
| **O1285** | 5'-TTTTTTGGATCCATGGCTCCATCTGCTAAGGCTACTGCCGCTAAG-3' |
| **O1287** | 5'-TTTTTTGGATCCATGGCTAAGTTCTTGAAAGCTGGTAAAGTTGCTGTCGTTGTCCGTGGTCGT -3' |
| **O1288** | 5'-TTTTTTGGATCCATGCCTTCCAGATTCACTAAG-3' |
| **O1289** | 5'-TTTTTTGGATCCATGGCTGAATCCCATAGATTGTACGTCAAAGGTAAGCAC-3' |
| **O1290** | 5'-TTTTTTGGATCCATGGCTCAACGTGTTACTTTC-3' |
| **O1291** | 5'-TTTTTTGGATCCATGGCCGGTGTTAAAGCTTACG-3' |
| **O1294** | 5'-TTTTTTCTGCAGAGCGCCTTATAATCATTGCTG-3' |
| **O1295** | 5'-TTTTTTCTGCAGTGACCCGTTTAATAGAATGCC-3' |
| **O1297** | 5'-TTTTTTCTGCAGTTAAACAACAACACCAGAGTTG-3' |
| **O1298** | 5'-TTTTTTCTGCAGTTAAGCGATCAATTCAACAAC-3' |
| **O1299** | 5'-TTTTTTCTGCAGTTATTTCTTAGACTTCTTTTC-3' |
| **O1300** | 5'-TTTTTTGGATCCATGGCTAGATACGGTGCTAC-3' |
| **O1302** | 5'-TTTTTTCTGCAGTTAAGCAGCAATACGCTTTTG-3' |
| **O1384** | 5'-TTTTTTGGATCCGCACTAGTTCCCAGGAAACCC-3' |
| **O1385** | 5'-ACTTTCGCATGGGTCAATACC-3' |
| **O1386** | 5'-TTTTTTGGATCCGTTCTTTATTTGTTGCCGC-3' |
| **O1387** | 5'-TTTTTTCTGCAGTTGAACGTATGTATGAATG-3' |
| **O1388** | 5'-TTTTTTGGATCCTTGTAAGACTGTTTTCCAG-3' |
| **O1389** | 5'-TTTTTTCTGCAGGATCCGTGGTTCATTAAAGA-3' |
| **O1396** | 5'-TTTTTTGGATCCGAATATTGCGTATAAGGAC-3' |
| **O1397** | 5'-TTTTTTCTGCAGACCAATTTTACACGATGCC-3' |
| **O1398** | 5'-TTTTTTGGATCCGCAATATTATACTTGTTAG-3' |
| **O1399** | 5'-TTTTTTCTGCAGGTGAAGTTAGACTTTTACC-3' |
| **O1404** | 5'-TTTTTTGGATCCATGAGCCAAACGAGGCTGAGA-3' |
| **O1405** | 5'-TTTTTTCTGCAGGGCTGGATATACTACTGAG-3' |
| **O1412** | 5'-TTTTTTGGATCCTTATTGACTGCAATTTGAG-3' |
| **O1413** | 5'-TTTTTTCTGCAGCAACCAAGAATAGTAAGAG-3' |
| **O1424** | 5'-TTTTTTCTGCAGATACCACCGCTACGCGTTGAC-3' |
| **O1429** | 5'-TTTTTTGGATCCATGGCTCCAGGTAAGAAAGTTG-3' |
| **O1430** | 5'-TTTTTTGGATCCATGAAATACATCCAAACTGAAC-3' |
| **O1432** | 5'-TTTTTTGGATCCATGGCTAACTTGCGTACTCAAAAG-3' |
| **O1433** | 5'-TTTTTTGGATCCATGGGTAAATCACACGGTTACAGATCTCG-3' |
| **O1436** | 5'-TTTTTTGGATCCATGGCCTCCTTACCTCACCC-3' |
| **O1439** | 5'-TTTTTTGGATCCATGATCATTGAACCATCTTTGAAAG-3' |
| **O1441** | 5'-TTTTTTGGATCCAGATATATCTCTCTAACAATG-3' |
| **O1442** | 5'-TTTTTTGGATCCATGGCTAAAAGAACTAAGAAGG-3' |
| **O1443** | 5'-TTTTTTCTGCAGCGGGATGCCTAAGGTATATG-3' |
